# Adult-onset hippocampal α7 nicotinic acetylcholine receptor loss rapidly drives Alzheimer-like neuropathology in mice

**DOI:** 10.3389/fphar.2026.1929468

**Published:** 2026-09-07

**Authors:** Jun Li, Guanglin Liu, Xiaotong Wang, Zheng Song, Huan Chen, Hongwei Hou, Qingyuan Hu

**Affiliations:** 1 Anhui Institute of Optics and Fine Mechanics, Hefei Institutes of Physical Science, Chinese Academy of Sciences, Hefei, China; 2 University of Science and Technology of China, Hefei, China; 3 Beijing Life Science Academy, Beijing, China

**Keywords:** Alzheimer’s disease, amyloid-β, astrocyte, cognitive impairment, microglia, neuronal death, Tau, α7 nAChR

## Abstract

**Introduction:**

The reduction of α7 nicotinic acetylcholine receptor (nAChR) in the hippocampus con-stitutes one of the neuropathological features observed in the brains of individuals with Alzheimer’s disease (AD), and α7 nAChR is crucial to cognitive process. However, studies involving α7 nAChR knockout mice have not demonstrated any abnormal brain structure or damage to hippocampal neurons, a discrepancy likely driven by widespread developmental compensatory signaling reprogramming triggered by permanent whole-body α7 nAChR deletion from embryonic stages. This limitation renders traditional knockout lines unable to recapitulate the adult-onset, hippocampus-restricted α7 nAChR insufficiency characteristic of human AD, creating an unaddressed knowledge gap regarding whether isolated α7 nAChR loss in mature hippocampal neurons independently drives AD-related neurodegeneration and cognitive dysfunction.

**Methods:**

To resolve this limitation, we established a spatially and temporally restricted knockdown mouse model *via* stereotaxic intrahippocampal injection of adeno-associated virus encoding *Chrna7*-targeted short hairpin RNA (shRNA) in 4-month-old mice. We assessed behavioral performance *via* open field test, novel object recognition test, Y-maze and Barnes maze 1 month post-viral delivery to quantify cognitive function; 2 months after injection, we harvested brain tissues to measure Aβ deposition, tau hyperphosphorylation, neuronal loss, astrocytic and microglial activation using immunofluorescence staining, and performed transcriptome RNA sequencing to profile genome-wide transcriptional alterations.

**Results:**

The knockdown of α7 nAChR in the hippocampus of adult mice rapidly induced cognitive deficits, impairments in learning and memory, and led to the increase of amyloid-β levels, Tau protein aggregation, neuronal damage, and the activation of astrocytes and microglia. Transcriptomic analysis revealed that the differentially expressed genes were significantly enriched in pathways related to inflammatory signaling.

**Discussion:**

Our study demonstrates that selective depletion of hippocampal α7 nAChR in adult mice is sufficient to trigger a spectrum of AD-like pathological alterations. Importantly, our AAV-mediated region-specific knockdown paradigm fully eliminates the developmental compensatory bias inherent to constitutive global α7 nAChR knockout animals by restricting receptor suppression exclusively to mature hippocampal neurons in post-developmental mice, closely recapitulating the spatial-temporal pattern of α7 nAChR loss in human AD brains. Our work provides novel pharmacological insights into α7 nAChR as a promising therapeutic target for AD pathological intervention.

## 1 Introduction

Alzheimer’s disease (AD), a neurodegenerative disorder and the leading cause of dementia, is characterized by the progressive decline in memory, cognitive function, and behavior (Koenig et al., 2016). The pathogenesis of AD is complex and remains unclear. The cholinergic hypothesis, one of the earliest theories proposed to elucidate the pathogenesis of AD, suggests that damage to cholinergic neurons in the basal forebrain of AD patients result in a reduction in acetylcholine levels, consequently leading to pathological features such as cognitive impairment (Hampel et al., 2018). However, in patients with mild cognitive impairment and early AD, the degeneration of cholinergic neurons in the basal forebrain is not prominently observed (Gilmor et al., 1999). Treatment with cholinesterase inhibitors to elevate acetylcholine levels merely slows disease progression without halting it, implying that postsynaptic signalling mechanisms underlying cholinergic transmission may be impaired in AD (Sarter and Bruno, 2002). In fact, the damage of nicotinic acetylcholine receptors (nAChR) is an early phenomenon of AD (Nordberg, 2001). As a pivotal structure for learning and memory, the hippocampus receives a major source of acetylcholine from cholinergic neurons located in the basal forebrain (Schliebs and Arendt, 2011), which plays a crucial role in emotion and cognition (Mineur et al., 2013; Zhang et al., 2016). Concurrently, in AD patients, there is a marked reduction in the expression of the α7 nAChR in the hippocampus (Guan et al., 2000), which may contribute to the cholinergic system-related cognitive impairment observed in AD.

The nAChR family comprises various subtypes, among which the α7 nAChR is one of the most abundant subtypes in the brain (Toyohara et al., 2010). Cholinesterase inhibitors and α7 nAChR agonists have been shown to alleviate symptoms in AD patients and animal models to varying extents (Fan et al., 2015; Ogos et al., 2024), highlighting the significance of α7 nAChR in AD. As an example, the α7 nAChR agonist cotinine improves learning and memory, mitigates Aβ pathology and reduces tau hyperphosphorylation in AD animal models (Echeverria and Zeitlin, 2012; Echeverria et al., 2011; Grizzell et al., 2017; Patel et al., 2014). However, in α7 nAChR knockout mice, no abnormal brain structure or hippocampal neuron damage was observed (Cordero-Erausquin et al., 2000). Furthermore, various studies on AD model mice have demonstrated that the knockout of the α7 nAChR gene can have opposing effects, either ameliorating or exacerbating AD pathology (Dziewczapolski et al., 2009; Hernandez et al., 2010). Interestingly, a recent study reported that α7 nAChR knockout mice exhibited synaptic plasticity and memory impairments in the hippocampus at 12 months of age, along with elevated amyloid precursor protein expression and increased levels of Aβ (Tropea et al., 2021). Of note, given the widespread expression of α7 nAChR throughout the human body and its involvement in numerous physiological functions, the non-tissue-specific and non-developmental stage-specific knockout of α7 nAChR may result in systemic and non-specific interference, complicating the interpretation of the results (Palma et al., 2012). Additionally, the potential functional compensation mechanism within the gene knockout model may result in an unchanged phenotype (Kim et al., 2015; Luzuriaga-Neira et al., 2022; Velazquez et al., 2018), which could account for the absence of associated phenotypes in α7 nAChR gene knockout mice (Tropea et al., 2021). Consequently, studies utilizing gene knockout models may lead to the misconception that α7 nAChR is not significant in AD, or that only individuals congenitally deficient in α7 nAChR develop AD-like pathology. In reality, the loss of α7 nAChR in AD patients predominantly occurs in the hippocampus of adults (Guan et al., 2000), and previous research employing α7 nAChR gene knockout mice does not appear to accurately reflect this phenomenon.

Recombinant adeno-associated virus (rAAV)-driven genetic manipulation offers a viable solution to the developmental compensation confounding traditional gene knockout murine models (Velazquez et al., 2018). This strategy enables temporally controlled, hippocampus-restricted suppression of α7 nAChR exclusively in mature adult mice, recapitulating the late-onset, region-specific α7 nAChR loss observed in sporadic AD patients, and avoid lifelong pan-tissue gene deletion. Although previous studies have reported the feasibility of specifically knocking down the expression level of α7 nAChR in adult mouse hippocampus through rAAV, the actual knocking-down efficiency has not been verified in mouse hippocampus, and the consequences of α7 nAChR knocking-down have not been described (Puliatti et al., 2026). To accurately assess the effects of α7 nAChR loss in the adult brain while circumventing potential compensatory mechanisms inherent in gene knockout models, we employed a rAAV engineered to express a short hairpin RNA specifically targeting α7 nAChR. By administering this rAAV into the hippocampus, we achieved a more precise simulation of the α7 nAChR deficiency observed in AD patients. We observed that in 4-month-old wild-type mice, the decrease of α7 nAChR level in hippocampus rapidly induces an AD-like phenotype.

## 2 Materials and methods

### 2.1 Animals

C57BL6 male mice at 4 months of age were purchased from Cyagen Bioscience Inc. (Suzhou, China). All mice were maintained in specific pathogen-free (SPF) condition with free access to food and water and were housed in four to five littermates with a 12-h/12-h reversed light/dark cycle, constant temperature (22 °C ± 2 °C) and humidity (50% ± 10%). This study underwent review and received approval from the China National Tobacco Quality Supervision and Test Center (Approval Number: CTQTC-SYXK-2024008). All animals involved in our research were cared under the provision and suggestions of the Chinese Experimental Animals Administration Legislation.

### 2.2 Virus vectors

The rAAV2/9-U6-shRNA (Chrna7)-CMV-EGFP and rAAV2/9-U6-shRNA (scramble)-CMV-EGFP were purchased from BrainVTA (Wuhan, China). The target sequence for the rAAV-shRNA (Chrna7) is GCC​TAA​GTG​GAC​CAG​GAT​CAT. To exclude off-target effects, the rAAV9-U6-shRNA (Chrna7)-CMV-EGFP and rAAV9-U6-shRNA (scramble)-CMV-EGFP were purchased from BrainCase (Shenzhen, China). The target sequence for the second rAAV-shRNA2 (Chrna7) is GCA​GTG​GAA​CAT​GTC​TGA​GTA​TTC​AAG​AGA​TAC​TCA​GAC​ATG​TTC​CAC​TGC. The target sequence for the rAAV-shRNA (scramble), which serves as a non-target shRNA control, is CCT​AAG​GTT​AAG​TCG​CCC​TCG. According to the manufacturer’s validation report, both sequences reduced *Chrna7* mRNA levels by more than 80% when transfected into 293T cells overexpressing mouse *Chrna7*.

### 2.3 Antibodys and reagents

The rabbit antibody against p-Tau (Thr181) (Cat# 12885, RRID: AB_2798053) was purchased from Cell Signaling Technology (Danvers, MA, United States). The mouse antibody against Aβ (4G8) (Cat# 800709, RRID: AB_2565325) was purchased from Biolegend (San Diego, CA, United States). The rabbit antibody against NeuN (Cat# GB150143, No RRID currently assigned) and Aβ1-40 (Cat# GB150027, No RRID currently assigned) were purchased from Servicebio (Wuhan, Hubei, China). The rabbit antibody against α7 nAChR (Cat# 82848-3-RR, RRID: AB_3670574), APP (Cat# 25524-1-AP, RRID: AB_2880118), GFAP (Cat# 16825-1-AP, RRID: AB_2109646) were purchased from Proteintech (Wuhan, Hubei, China). The rabbit antibody against BACE1 (Cat# ab183612, RRID: AB_3094757), Iba1 (Cat# ab178847, RRID: AB_2832244), GAPDH (Cat# ab181602, RRID: AB_2630358) were purchased from Abcam (Cambridge, United Kingdom). The goat anti-mouse IgG secondary antibody conjugated to Alexa Fluor (AF) 647 (Cat# A-21235, RRID: AB_2535804), goat anti-mouse IgG secondary antibody conjugated to AF555 (Cat# A-21422, RRID: AB_2535844), goat anti-rabbit IgG secondary antibody conjugated to AF647 (Cat# A-21244, RRID: AB_2535812), goat anti-rabbit IgG secondary antibody conjugated to AF647 (Cat# A-21428, RRID: AB_2535849) were purchased from Thermo Fisher Scientific (Waltham, MA, United States). The HRP-linked anti-rabbit IgG secondary antibody (Cat# 7074, RRID: AB_2099233) was purchased from Cell Signaling Technology (Danvers, MA, United States). Hoechst 33,342 (Cat# C1022) was purchased from Beyotime (Shanghai, China).

### 2.4 Stereotactic viral delivery

To reduce α7 nAChR expression in hippocampus, rAAV-U6-shRNA (*Chrna7*)-CMV-EGFP-SV40 pA virus was injected into the hippocampus of male mice at the age of 4 months. This virus specifically targets the Chrna7 gene, which encodes the α7 nAChR. Mice were anesthetized with intraperitoneal injection of tribromoethanol (125–250 mg/kg) and immobilized in a stereotactic injection apparatus (RWD Life Science, Shenzhen, China). After the scalp was disinfected and balanced, a tiny hole was punched very gently. A glass pipette filled with the viral vector was attached to a microsyringe pump (RWD, Shenzhen, China). The rAAV-shChrna7 (α7 KD group) or scramble shRNA control rAAV (control group) was infused bilaterally into both the dorsal and ventral hippocampus at the following stereotaxic coordinates: dorsal hippocampus, AP: −1.94 mm, ML: ±1.25 mm, DV: −1.8 mm; ventral hippocampus, AP: −3.4 mm, ML: ±2.75 mm, DV: −3.5 mm. The rAAVs, with a viral titer of 5.38 × 10^12^ vg/mL, were delivered at a rate of 60 nL/min, with a volume of 100 nL per injection site. Following the injection, the needle was retained in position for an additional 3 minutes before being gradually withdrawn. The second rAAV-shRNA2, with a viral titer of 2.5 × 10^12^ vg/mL, was utilized for a separate unilateral internal control experiment restricted exclusively to the dorsal hippocampus (AP: −1.94 mm, ML: ±1.25 mm, DV: −1.8 mm). For this paradigm, each mouse received rAAV-shChrna7 in the left dorsal hippocampus and scramble shRNA rAAV in the contralateral right dorsal hippocampus, with a total injection volume of 200 nL per injection site.

### 2.5 Behavioral test

Mice were habituated to the testing room for 2 h before behavioral experiments to minimize stress-induced variability. The animals’ behavior was recorded automatically using a camera positioned above the behavioral detection platform and subsequently analyzed with video tracking software (Visutrack, Xinruan Information Technology Company, Shanghai, China).

The open field test was performed to assess exploratory activity and anxiety. Mice were individually placed in a square open field (40 cm × 40 cm) and allowed to freely explore for 10 min while the trial was videotaped. The open-field arena was averagely divided into 16 blocks. The outer zone was composed of 12 blocks, and the center zone was composed of four blocks.

The novel object recognition test was used to evaluate the long-term cognitive abilities of the animals. For two consecutive days, the mice were allowed to get acquainted with two identical objects placed in the apparatus (40 cm × 40 cm) for 10 min. On the third day, one of the two familiar objects was replaced with a novel object, and the mice were gently placed in the apparatus and allowed to explore the objects for 10 min while the trial was videotaped. The exploration was defined as sniffing (within 1 cm), biting, or pawing. The number of explorations of familiar objects (F) and novel objects (N) was analyzed. The recognition index (RI) was calculated as the formula: RI = N/(N + F) ×100%.

The Y maze test was used to evaluate the short-term spatial memory. Each mouse was placed at the end of one arm of the Y-maze and allowed to explore freely for 8 min. Spontaneous alternations were defined as entries into all three arms on consecutive occasions. The spontaneous alternation rate (%) = [number of valid alternations/(total arm entries −2)] × 100.

The Barnes maze was used to evaluate the spatial memory and learning. The apparatus consisted of a round platform with a diameter of 75 cm, elevated 60 cm above the floor. The maze featured twenty holes, each with a diameter of 5 cm, positioned 5 cm from the perimeter. A black escape box was situated beneath one of these holes. Distinct spatial cues were strategically placed around the maze and remained constant throughout the duration of the study. On the day preceding the test, the mice underwent a training session in which they were allowed to explore the platform freely for 4 min, followed by placement in the escape box for 1 minute. At the commencement of each phase, the mice were situated in a cylindrical black starter chamber, 10 cm in height, positioned at the center of the maze. After a period of 5 s, the starter chamber was removed, and both an auditory stimulus (buzzer at 80 dB) and a visual stimulus (light at 400 lux) were activated, allowing the mice to explore the maze autonomously. The trial concluded either when the mouse entered the escape passage or after a duration of 4 min. Upon entry into the escape passage, the buzzer was deactivated, and the mouse was permitted to remain in darkness for 1 min. If the mouse did not independently enter the tunnel, it was gently put in the escape box for 1 min. Mice were tested once a day for 5 days for the acquisition portion of the study.

The rotarod test was used to evaluate the motor ability. Mice were trained on the rotarod for three consecutive days, during which the rod accelerated from 4 to 40 rpm over 5 min. All animals were tested three times with at least a 5 min break between tests, and the mean latency to fall was documented.

### 2.6 Immunofluorescence staining

Brains were harvested quickly without perfusion. The brains were fixed in 4% paraformaldehyde diluted in phosphate buffered saline (PBS, 2.7 mM KCl, 2.0 mM KH_2_PO_4_, 137 mM NaCl, 10 mM Na_2_HPO_4_, pH 7.3–7.5) at 4 °C for 24 h. For cryoprotection, fixed mouse brains underwent gradient sucrose dehydration at 4 °C. Tissues were first incubated in 15% sucrose diluted in PBS for approximately 12 h until they sank entirely. The solution was then exchanged for 30% sucrose in the same PBS buffer, and tissues were incubated for approximately 24 h until full submersion. After sucrose dehydration, brains were snap-frozen over liquid nitrogen vapor and then embedded in OCT and frozen at −20 °C. Serial coronal brain sections with a thickness of 20 μm were cut using a cryostat microtome. Continuous serial slices covering the entire dorsal and ventral hippocampus were collected. PBST was prepared by adding 0.05% (v/v) Tween-20 to PBS. Sections were permeabilized and blocked in PBST containing 0.3% Triton X-100 and 5% normal goat serum for 1 h at room temperature. The cells were then incubated with the primary antibodies overnight at 4 °C. The following day, sections were incubated with secondary antibodies overnight at 4 °C in dark. Nuclei were stained with Hoechst 33,342. Primary antibodies against Aβ (4G8), Aβ1-40 and Iba1 were diluted at 1:200, whereas antibodies targeting p-Tau, NeuN and GFAP were diluted at 1:500 in PBST supplemented with 0.3% Triton X-100 and 1% BSA as the antibody diluent. Samples were mounted on coverslip with antifade mounting medium (Cat# PH0429, Phygene, Fuzhou, China). All slides were scanned using a digital scanner (PANNORAMIC MIDI II, 3DHISTECH). Three discontinuous sections were analyzed per mouse, and the mean value per mouse is represented as a dot. Images were captured at ×20 objective. The mean fluorescence intensity (MFI), particle count, or positive area within the hippocampus was analyzed using ImageJ software.

### 2.7 Western blotting

Total proteins were extracted using RIPA lysis buffer. In total, 20 μg of total proteins were separated using 12% SurePAGE (Cat# M00669, GenScript, Nanjing, China), and transferred to polyvinylidene fluoride (PVDF) membranes. The membranes were blocked with TBST (10 mM Tris, 150 mM NaCl, 0.05% Tween-20, pH 7.2–7.6) containing 5% skim milk powder for 1 h at room temperature, and were incubated with primary antibodies overnight at 4 °C on a shaker. Following a washing step with TBST, membranes were incubated with HRP-conjugated secondary antibodies for 1 h at room temperature. All target primary antibodies and HRP-conjugated secondary antibodies were diluted at 1:1,000 in TBST containing 5% BSA, and anti-GAPDH antibody was diluted at 1:10,000 in the same buffer. The protein bands were finally visualized using the Western BLoT Hyper HRP Substrate (Cat# T7103A, Takara, Tokyo, Japan), and their images were acquired using a Gel Imaging System (Servicebio, Wuhan, China). The gray value of protein bands was analyzed using ImageJ. The gray value ratio of target protein to internal reference (GAPDH) was regarded as the relative protein expression. All original WB results are shown in Supplementary Material.

### 2.8 TUNEL staining

TUNEL staining was performed using a Tunel Cell Apoptosis Detection Kit (Cat# G1502, Servicebio, Wuhan, China). Samples were prepared according to the manufacturer’s manual. Briefly, sections were washed with PBS and were treated with 20 μg/mL Proteinase K for 10 min. For positive control slices, additional treatment with DNase I (20 U/mL, 10 min) was conducted prior to TdT incubation. Followed by incubation with TdT working solution for 1 h at 37 °C.

### 2.9 Detection of cytokines

The hippocampus was homogenized in ice-cold saline to prepare 10% homogenates. The levels of cytokines and chemokines in hippocampal homogenates were assayed by multiplexed bead array immunoassay using Bio-Plex Pro Mouse Cytokine 23-plex Assay Kit according to the manufacturer’s protocol (Cat# M60009RDPD, Bio-Rad, Hercules, CA, United States).

### 2.10 Quantitative RT-qRCR analysis

Total RNA from mouse hippocampus was isolated using an RNA extraction kit (Cat# AG21017, Accurate Biology, Changsha, China). RNA from each sample was reverse transcribed into cDNA by an Evo M-MLV RT Mix Kit (Cat# AG11728, Accurate Biology, Changsha, China). Subsequently, RT-qPCR was performed on Lightcycler 96 System (Roche Diagnostics, Mannheim, Germany) using SYBR Green Premix Pro Taq HS qPCR Kit (Cat# AG11728, Accurate Biology, Changsha, China). We calculated the relative expression levels of target genes based on the 2^−ΔΔCT^ method. qPCR primers are summarized as follows: Chrna7: forward, 5′- CCA​GTA​TCT​CCC​TCC​AGG​CAT -3′ and reverse, 5′- AGG​ACC​ACC​CTC​CAT​AGG​AC -3’; Actb: forward, 5′- GGC​TGT​ATT​CCC​CTC​CAT​CG -3′ and reverse, 5′- CCA​GTT​GGT​AAC​AAT​GCC​ATG​T -3’.

### 2.11 RNA sequencing and data analysis

The sequencing service was provided by Novogene (Beijing, China). RNA was extracted, and its integrity was assessed using a Bioanalyzer 2100 system (Agilent Technologies, CA, United States). mRNA was purified using poly-T oligo-attached magnetic beads, and a library was prepared. Quantification and size distribution of the library were assessed using Qubit and real-time PCR, with size detection *via* a Bioanalyzer. The pooled libraries were subjected to Illumina sequencing. Raw fastq data were processed using fastp software, and reference genome and gene model annotation files were downloaded from the genome website. Reference genome indexing was performed using Hisat2 v2.0.5, and paired-end clean reads were aligned to the reference genome. FeatureCounts v1.5.0-p3 was used to count the reads mapped to each gene, and the FPKM of each gene was calculated based on gene length and read count. Differential gene expression analysis was performed with DESeq2 software. Genes with |log2fold-change| ≥ 1 and padj <0.05 were defined as differentially expressed genes (DEGs). The Gene Ontology (GO) and Kyoto Encyclopedia of Genes and Genomes (KEGG) pathway enrichment analysis was performed by clusterProfiler. The Gene Set Enrichment Analysis (GSEA) was generated using the online platform available at https://www.bioinformatics.com.cn, which specializes in data analysis and visualization. For the analysis of protein-protein interaction (PPI), the initial step involves utilizing the Search Tool for the Retrieval of Interacting Genes/Proteins (STRING) to ascertain the PPI association. Subsequently, based on the established PPI associations, a PPI network is constructed employing the Cytoscape software platform (version 3.10.4).

### 2.12 Statistics

Normality was tested by Shapiro-Wilk Normality test. Unpaired Student’s t-test or paired *t*-test was used for the statistical analyses. The nonparametric Mann-Whitney *U* test was adopted for groups with fewer than five samples. Barnes maze latency data were subjected to two-way repeated-measures ANOVA (group × training day) and Greenhouse–Geisser sphericity correction was applied. Upon detection of a significant group × day interaction, Bonferroni-corrected simple effect comparisons were performed separately at each training day. Two mice with incorrect virus injections were excluded from the analysis. *P* < 0.05 was considered as statistically significant.

## 3 Results

### 3.1 Construction of hippocampal α7 nAChR knockdown *via* rAAV-shRNA

**FIGURE 1:**
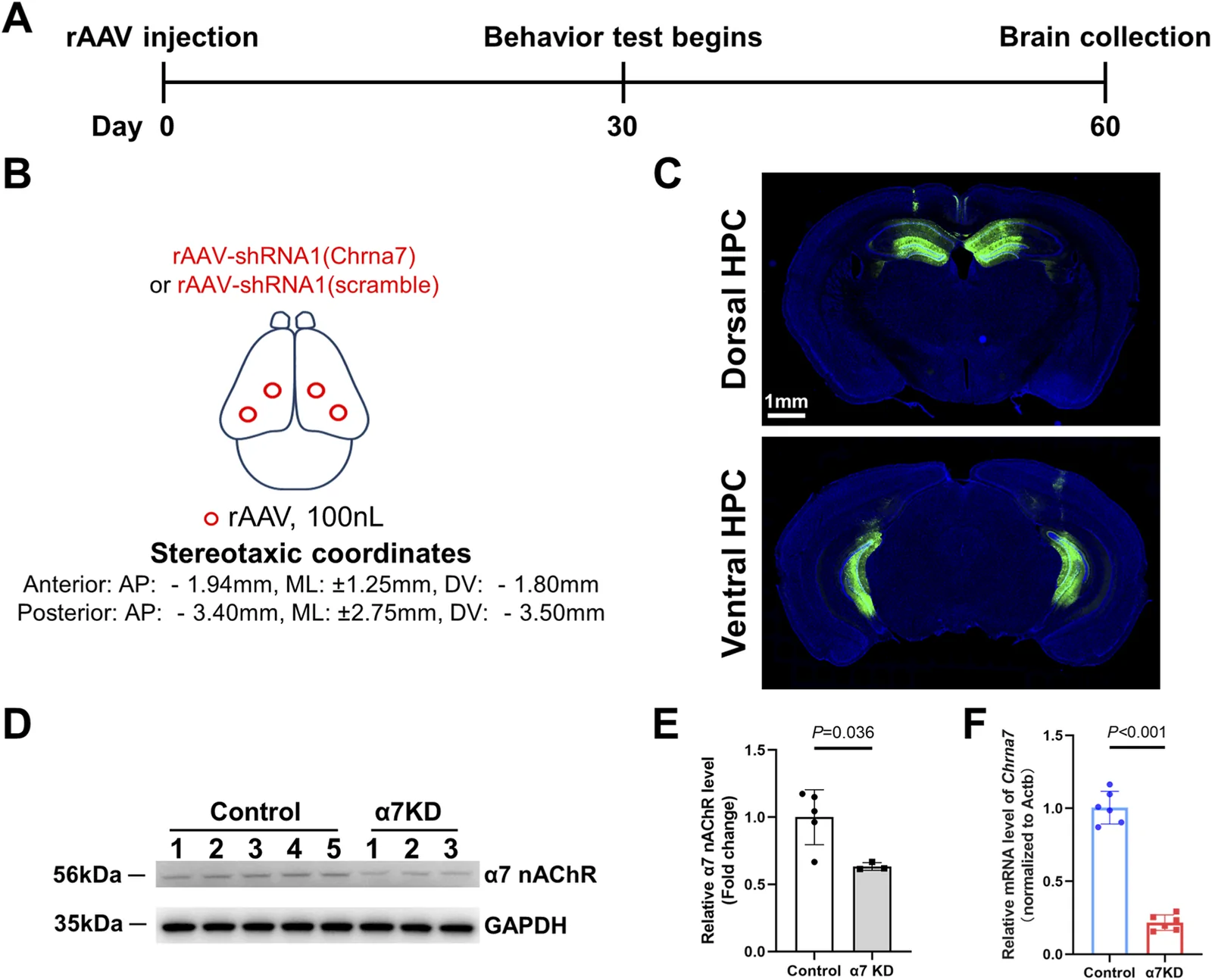
rAAV-shChrna7 mediates hippocampal α7 nAChR knockdown. **(A)** Diagram of the experimental design. **(B)** Schematic of rAAV injection in 4-month-old mice. **(C)** Representative EGFP fluorescence images showing the injection sites of control rAAV-scramble shRNA in mouse hippocampus. **(D)** Western blot of hippocampus lysates using α7 nAChR antibody. **(E)** Quantitative analysis of western blot in **D**, *n* = 5 control mice and 3 α7 KD mice, *P* values were determined by Mann-Whitney *U* test. **(F)** The relative mRNA expression of *Chrna7* in hippocampal tissues of control and α7 KD mice was quantified by RT-qPCR (*t* = 15.56, df = 10), *n* = 6 control mice and 6 α7 KD mice, *P* values were determined by Student’s t-test. Data are presented as the mean ± SD. HPC, hippocampus.

In order to reduce α7 nAChR expression in hippocampus, we used a recombinant adeno-associated virus (rAAV)-U6-short hairpin (sh)RNA (*Chrna7*)-CMV-EGFP-SV40 pA to interfere with α7 nAChR expression. Experimental timelines are shown in Figure 1A. Behavioral tests were conducted 30–60 days post-injection, and the animals were sacrificed on the 60th day. Each animal received bilateral stereotaxic injections at four symmetric hippocampal sites in total: one dorsal and one ventral target per hemisphere, with identical coordinates used for the left and right hippocampus. Each site receiving 100 nL, totaling 400 nL of rAAV per mouse (Figure 1B). The injection sites were verified by fluorescence imaging (Figure 1C). Western blot showed the reduced level of α7 nAChR (*P* = 0.036) (Figures 1D,E). RT-qPCR analysis on hippocampal tissues harvested at 1 month post rAAV injection further confirmed the altered Chrna7 expression (*P* < 0.001) (Figure 1F). Taken together, the above evidence demonstrates that stereotaxic delivery of rAAV-shChrna7 achieves robust downregulation of hippocampal α7 nAChR at transcriptional and translational levels.

### 3.2 Acute α7 nAChR knockdown in hippocampus impaired spatial learning and memory

**FIGURE 2:**
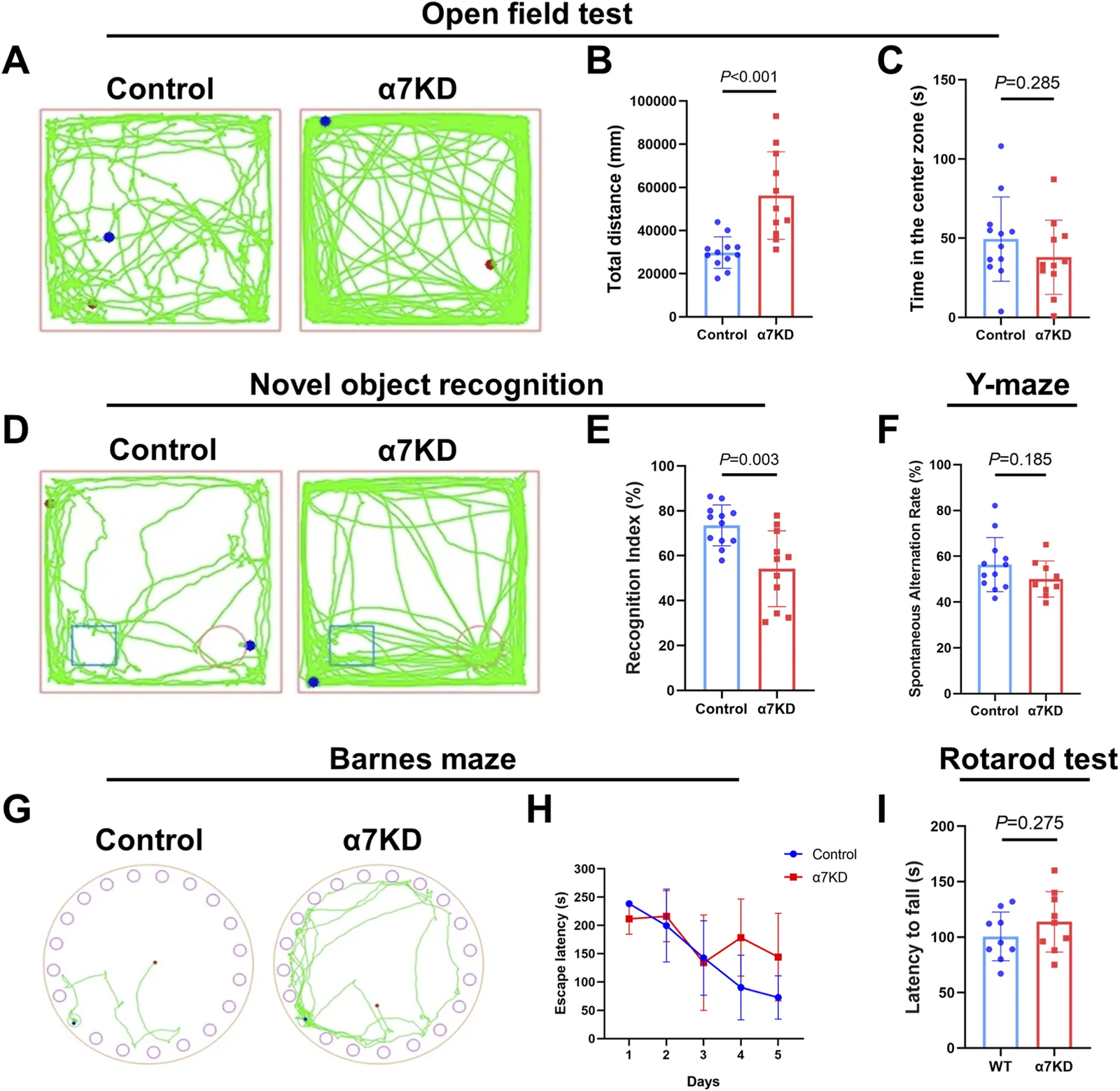
Acute α7 nAChR knockdown in hippocampus leads to spatial learning and memory dysfunction. **(A)** Representative traces of mouse in the open field test. Red dots indicate the mouse’s starting point and blue dots indicate the final position of the mouse. **(B)** Total distance travelled during 10 min of open field test (*t* = 4.229, df = 21), *n* = 12 control mice and 11 α7 KD mice. **(C)** Time spent in the centre zone of the open field during 10 min of open field test (*t* = 1.097, df = 21), *n* = 12 control mice and 11 α7 KD mice. **(D)** Representative tracing path in the novel object recognition test. Red circles and blue squares represent familiar and novel objects, respectively. **(E)** Recognition index of the mice in novel object recognition test (*t* = 3.448, df = 21), *n* = 12 control mice and 11 α7 KD mice. **(F)** Spontaneous alternation rate of the mice in Y-maze (*t* = 1.376, df = 19), *n* = 12 control mice and 9 α7 KD mice. **(G)** Representative tracing path in Barnes maze at day 5. **(H)** In Barnes maze, latency to reach the target hole is shown during training days, *n* = 10 control mice and 7 α7 KD mice. **(I)** The latency of the mice to fall from the rotarod (*t* = 1.131, df = 16), *n* = 9 mice per group. Data are presented as the mean ± SD. *P* values were determined by Student’s t-test.

To determine the effects of acute α7 nAChR knockdown on behavioral performance, we tested the mice in a series of cognitive and noncognitive behavioral tests on day 30. We used the open field test (OFT) to measure general motor function and anxiety-like behavior. Compared with the control mice, α7 nAChR knockdown (α7 KD) mice were hyperactive in the open field test, as indicated by increased total moving distance (*P* < 0.001) (Figures 2A,B). However, no significant difference was observed for the time spent in the center zone between the α7 KD and control mice (*P* = 0.285) (Figure 1C). The object recognition memory was assessed by using the novel object recognition test. The results showed that the new object recognition index of α7 KD mice decreased (*P* = 0.003) (Figures 1D,E), indicating impaired ability to distinguish new objects. Next, the Y maze test was performed to measure the short-term working memory. As shown in Figure 1F, α7 KD mice showed no impairment in spontaneous alternation behavior on the Y-maze (*P* = 0.185), suggesting intact working memory. We then tested the mice in Barnes maze, a spatial learning and memory task that require proper hippocampal function. Two-way repeated-measures ANOVA (between-subjects factor: group, consisting of α7 KD and Control groups; within-subjects repeated-measures factor: training day) identified a significant main effect of training day (*F*_(2.858, 42.88)_ = 19.27, *P* < 0.0001), indicating all mice gradually reduced escape latency with repeated training. The main effect of group did not reach statistical significance (*F*_(1, 15)_ = 2.238, *P* = 0.1554). Importantly, a significant group × training day interaction was detected (*F*_(4, 60)_ = 4.660, *P* = 0.0024), demonstrating distinct temporal learning profiles between Control and α7 KD mice. Although the global interaction indicated divergent learning trajectories, none of the individual day-wise pairwise comparisons reached statistical significance after Bonferroni correction (Day 1: *t* = 2.551, df = 6.320, adjusted *P* = 0.2074; Day 2: *t* = 0.6221, df = 14.99, adjusted *P* > 0.999; Day 3: *t* = 0.2196, df = 10.87, adjusted *P* > 0.999; Day 4: *t* = 2.802, df = 11.47, adjusted *P* = 0.0830; Day 5: *t* = 2.260, df = 8.073, adjusted *P* = 0.2673). This pattern suggests that the group difference manifests as a cumulative, gradual divergence across training rather than a robust difference at any single time point (Figures 1G,H). The accelerating rotarod test was used to evaluate motor function. The result showed that the motor function was comparable between control and α7 KD mice (*P* = 0.275) (Figure 1I). In conclusion, α7 KD mice exhibit significant spatial learning and memory deficits rather than impaired motor function.

### 3.3 Acute α7 nAChR knockdown in hippocampus induced Aβ pathology

**FIGURE 3:**
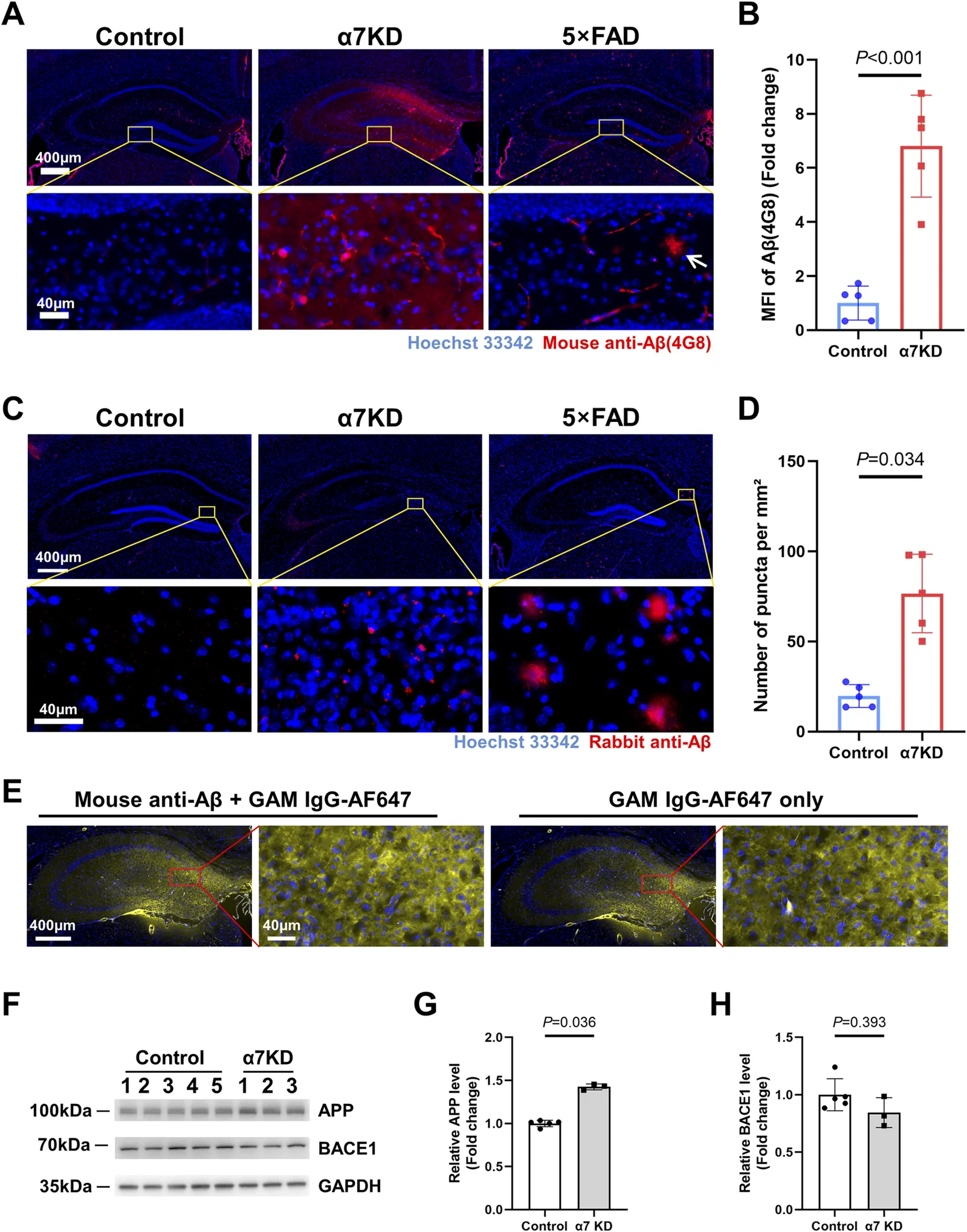
Acute α7 nAChR knockdown in hippocampus increases Aβ levels and APP expression. **(A)** Representative images of mouse anti-Aβimmunofluorescence staining. The white arrow denotes the Aβ classical plaque formed by human Aβ in 5xFAD mice. **(B)** Quantitative analysis of mean immunofluorescence intensity (MFI) in hippocampus in **A** (*t* = 6.541, df = 8), *n* = 5 control mice and 5 α7 KD mice. **(C)** Representative images of rabbit anti-Aβ antibody immunofluorescence staining. **(D)** Quantitative analysis of Aβ burden in hippocampus in C (*t* = 5.617, df = 8), *n* = 5 mice per group. For immunofluorescence, nuclei were counterstained with Hoechst 33,342 (blue). **(E)** Control staining to verify non-specific IgG signals in mouse brain tissue. Left panel: hippocampal sections incubated with mouse anti-Aβ primary antibody and goat anti-mouse fluorescent secondary antibody; Right panel: secondary-antibody-only negative control without primary antibody. GAM: goat anti-moue. **(F)** Western blot of hippocampus lysates using APP and BACE1 antibody. **(G)** Quantitative analysis of APP levels in F, *n* = 5 control mice and 3 α7 KD mice. **(H)** Quantitative analysis of BACE1 levels in **F**, *n* = 5 control mice and 3 α7 KD mice. Data are presented as the mean ± SD. Student’s t-test was used for **B and D**; Mann-Whitney *U* test was applied for **G and H**.

Aβ deposition is one of the neuropathological hallmarks of AD (Reiss et al., 2018). In order to verify the effect of acquired α7 nAChR deficiency on Aβ load in WT mice, we used two types of Aβ antibodies to detect the total Aβ load in hippocampus. Histological slices were treated by immunofluorescence staining with mouse or rabbit anti Aβ antibody to analyze the deposition of Aβ. As shown in Figures 3A–D, both anti-Aβ antibodies yielded valid specific staining signals in 5×FAD mice. However, abundant non-specific immunoreactivity was observed when using the mouse anti-Aβ antibody in α7 KD mice, whereas no such background staining was detected with the rabbit anti-Aβ antibody. Further staining with goat anti-mouse fluorescent secondary antibody confirmed that these non-specific signals originated from endogenous mouse IgG (Figure 3E). Accordingly, primary antibodies derived from mouse were excluded from all subsequent experiments. Compared with control mice, α7 KD mice exhibited a significantly elevated Aβ burden (*P* = 0.034) (Figures 3C,D). Notably, unlike the large amyloid plaques typically found in human APP-overexpressing 5×FAD mice, Aβ deposits in the hippocampus of α7 KD mice appeared as punctate granular aggregates rather than bulky plaques (Figure 3C). Western blot analysis of hippocampal lysates using anti-APP and anti-BACE1 antibodies was performed to characterize alterations in the Aβ production pathway. Compared with control mice, α7 KD mice exhibited markedly elevated APP expression (*P* = 0.036), while BACE1 levels remained unchanged (*P* = 0.393) (Figures 3F–H). Collectively, these data demonstrate that hippocampal α7 nAChR knockdown increases total Aβ burden and upregulates APP expression in 4-month-old mice.

### 3.4 Acute α7 nAChR knockdown in hippocampus induced Tau pathology

**FIGURE 4:**
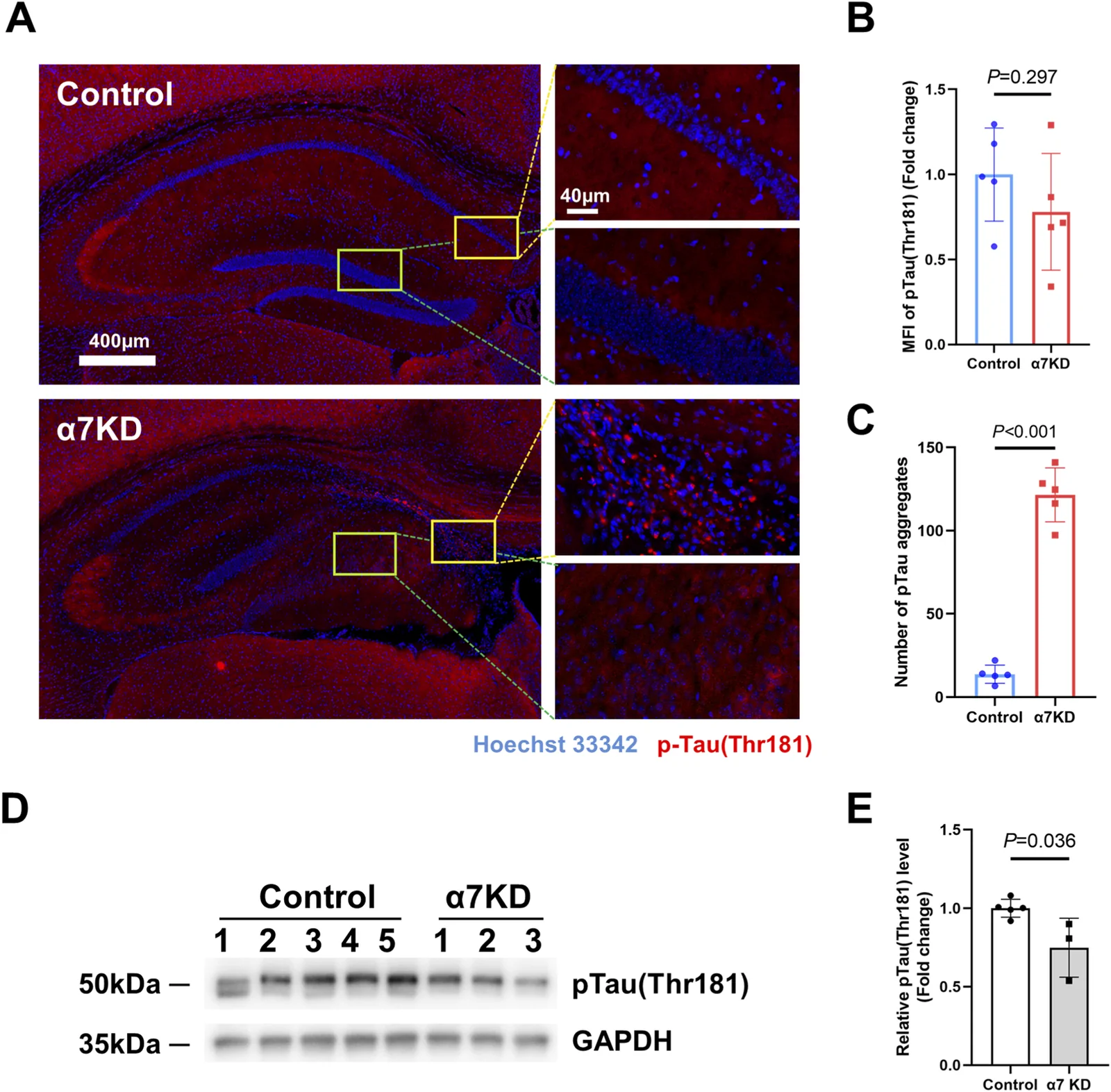
Acute α7 nAChR knockdown in hippocampus induces p-Tau aggregation. **(A)** The representative images of p-Tau (Thr181) immunofluorescence staining. **(B)** The MFI of p-Tau (Thr181) in hippocampus (*t* = 1.115, df = 8), *n* = 5 mice per group. **(C)** Quantification of p-Tau aggregates within the unilateral entire hippocampus per 20 μm-thick brain section (*t* = 14.11, df = 8), *n* = 5 mice per group. For immunofluorescence, nuclei were counterstained with Hoechst 33342 (blue). **(D)** Western blot of hippocampus lysates using p-Tau (Thr181) antibody. **(E)** Quantitative analysis of western blot in **D**, *n* = 5 control mice and 3 α7 KD mice. Data are presented as the mean ± SD. Student’s t-test was used for **B and C**; Mann-Whitney *U* test was applied for **E**.

Tau is hyper-phosphorylated in AD, with hyper-phosphorylation leading to Tau aggregation and subsequent pathology (Mamun et al., 2020). We detected the aggregation of phosphorylated Tau (p-Tau) at Thr181 in α7 KD mice by immunofluorescence. As shown in Figures 4A–C, compared with the control mice, there was no significant difference in the MFI of p-Tau in the hippocampus of α7 KD mice (*P* = 0.297). However, the number of p-Tau aggregates in the hippocampus of α7 KD mice increased significantly (*P* < 0.001), and these aggregates were mainly located in CA1 region. Notably, p-Tau aggregates were absent in the dentate gyrus, which exhibited severe morphological alterations, suggesting that these findings are unlikely to be attributed to the injection site. We also examined p-Tau level in hippocampus by western blot. The level of p-Tau decreased significantly (*P* = 0.036) (Figures 4D,E). These results showed that α7 nAChR knockdown caused p-Tau aggregation in hippocampus.

### 3.5 Acute α7 nAChR knockdown in hippocampus induced neuronal loss

**FIGURE 5:**
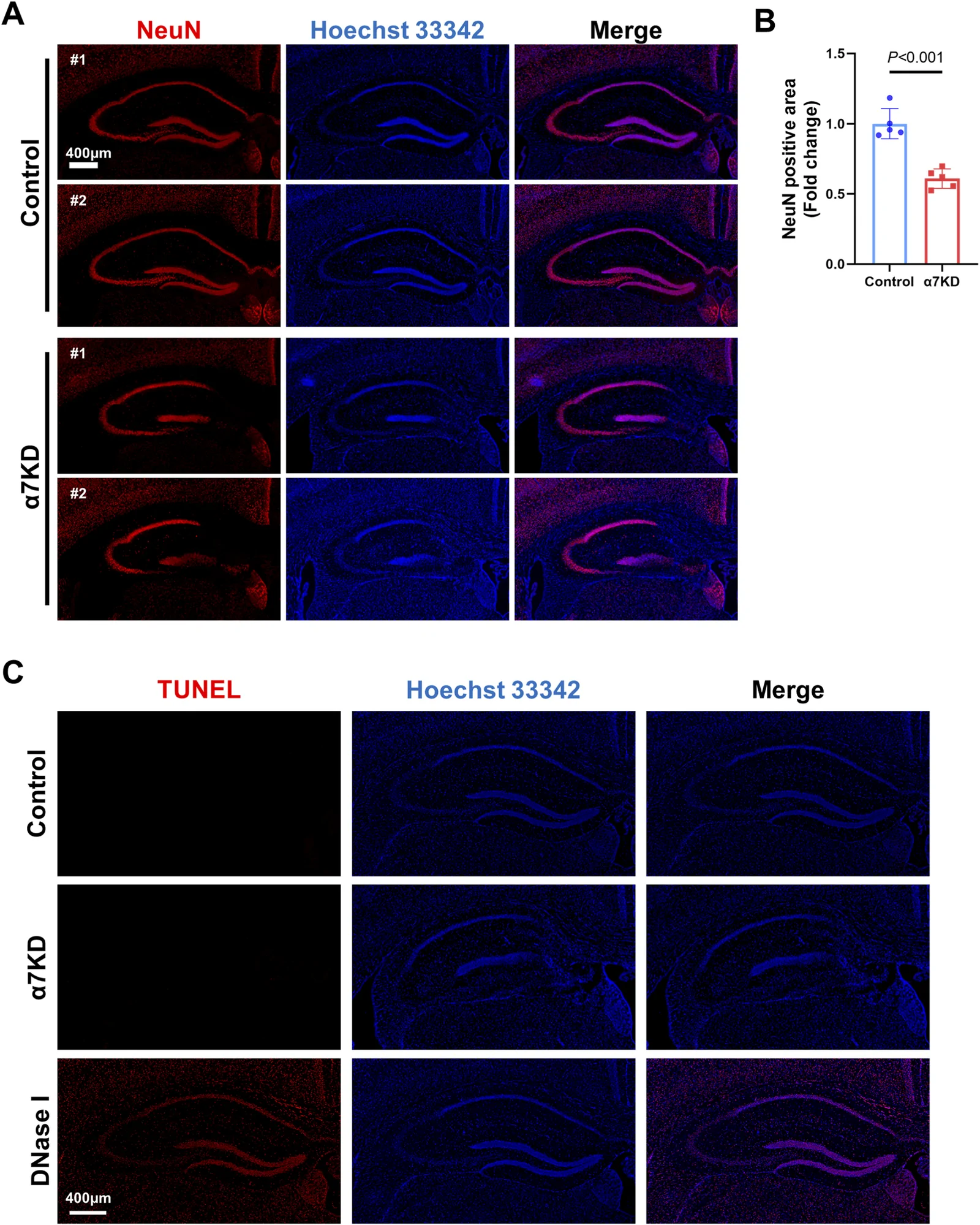
Acute α7 nAChR knockdown in hippocampus induces neuronal loss. **(A)** Representative images of NeuN immunofluorescence staining. **(B)** Quantification of the NeuN positive area in hippocampus (*t* = 6.835, df = 8), *n* = 5 mice per group. **(C)** Representative images of TUNEL staining. DNase I treatments were used as positive controls for TUNEL staining. Data are presented as the mean ± SD. *P* values were determined by Student’s t-test.

AD patients suffer from extensive selective neuronal loss in the hippocampal areas (Bobinski et al., 1997; Kril et al., 2002). To assess neuronal loss, neurons were labeled by the neuronal marker NeuN. The results showed that NeuN positive area in the hippocampus of α7 KD mice decreased significantly (*P* < 0.001) (Figures 5A,B), indicating the loss of neurons. Notably, neuronal loss is mainly found in hippocampal CA1 region and dentate gyrus, which is consistent with our virus injection site (Figure 1C). To further explore whether neuronal loss arose from apoptotic cell death, TUNEL staining was performed to label apoptotic neurons. However, barely detectable TUNEL-positive signals were observed in hippocampus in both control and α7 KD groups (Figure 5C). These results showed that α7 nAChR knockdown induced neuronal loss in hippocampus.

### 3.6 Acute α7 nAChR knockdown in hippocampus activated astrocytes and microglia

**FIGURE 6:**
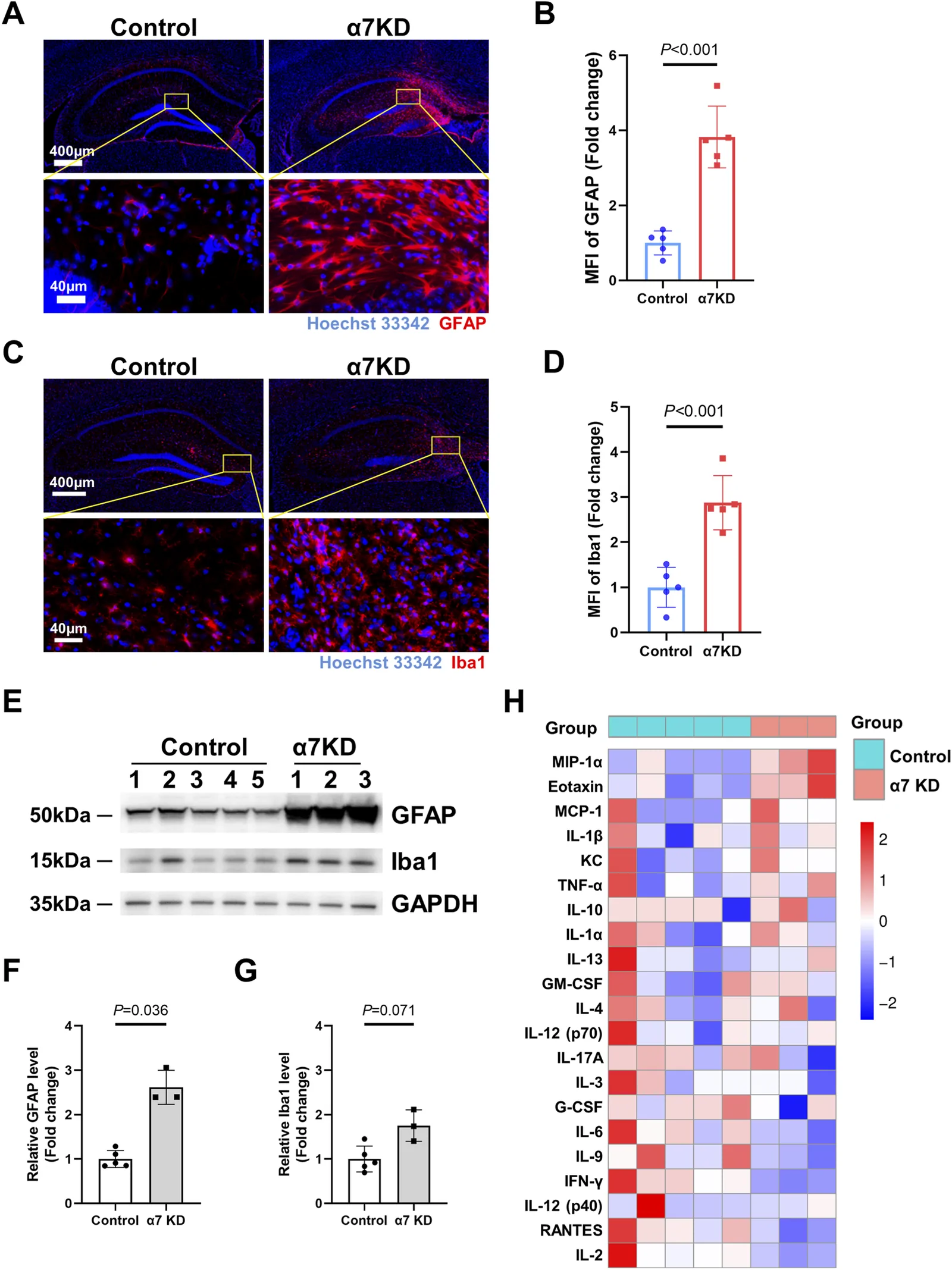
Acute α7 nAChR knockdown in hippocampus activates astrocytes and microglia. **(A)** Representative images of GFAP immunofluorescence staining. **(B)** The MFI of GFAP in hippocampus (*t* = 7.148, df = 8), *n* = 5 mice per group. **(C)** Representative images of Iba1 immunofluorescence staining. **(D)** The MFI of Iba1 in hippocampus (*t* = 5.621, df = 8), *n* = 5 mice per group. **(E)** Western blot of GFAP and Iba1 in the hippocampus. **(F)** Quantitative analysis of GFAP levels in **E**, *n* = 5 control mice and 3 α7 KD mice. **(G)** Quantitative analysis of Iba1 levels in **E**, *n* = 5 control mice and 3 α7 KD mice. **(H)** Heatmap visualization of 21 quantifiable inflammatory cytokines in mouse hippocampus, and all data were normalized by row Z-score. Data are presented as the mean ± SD. Student’s t-test was used for **B and D**; Mann-Whitney *U* test was applied for **F and G**.

Another feature of AD is the activation of astrocytes and microglia cells (Chen et al., 2025). The Immunofluorescence and Western blot was carried out to investigate the expression levels of GFAP, a marker of reactive astrocyte (Eng et al., 2000), and Iba-1, a marker of activated microglia (Ito et al., 1998). As shown in Figures 6A,B, the MFI of GFAP was significantly increased in the hippocampus of α7 KD mice (*P* < 0.001), indicating the activation of astrocytes. Similarly, the MFI of Iba1 was also significantly increased (*P* < 0.001) (Figures 6C,D), indicating the activation of microglia cells. Moreover, western blot analysis further confirmed these results (Figures 6E–G). To further verify the occurrence of neuroinflammation, a panel containing 23 inflammatory cytokines was quantified using the Bio-Plex multiplex assay. IL-5 and MIP-1β fell below the lower limit of quantification and were excluded from subsequent heatmap visualization. Among the remaining 21 detectable factors, 16 cytokines exhibited stable expression with no obvious upregulated or downregulated trends between groups, while only five cytokines displayed discernible differential expression tendencies (Figure 6H). Specifically, MIP-1α and Eotaxin showed an upward trend, whereas IFN-γ, RANTES and IL-2 were downregulated in α7 KD mice relative to controls. Collectively, these data imply that α7 nAChR knockdown in hippocampus induced activation of astrocytes and microglia cells. Meanwhile, the cytokine profiling suggested restricted inflammatory response rather than extensive neuroinflammation, with only several individual cytokines showing altered expression levels.

### 3.7 Acute α7 nAChR knockdown in hippocampus significantly affected transcriptome

**FIGURE 7:**
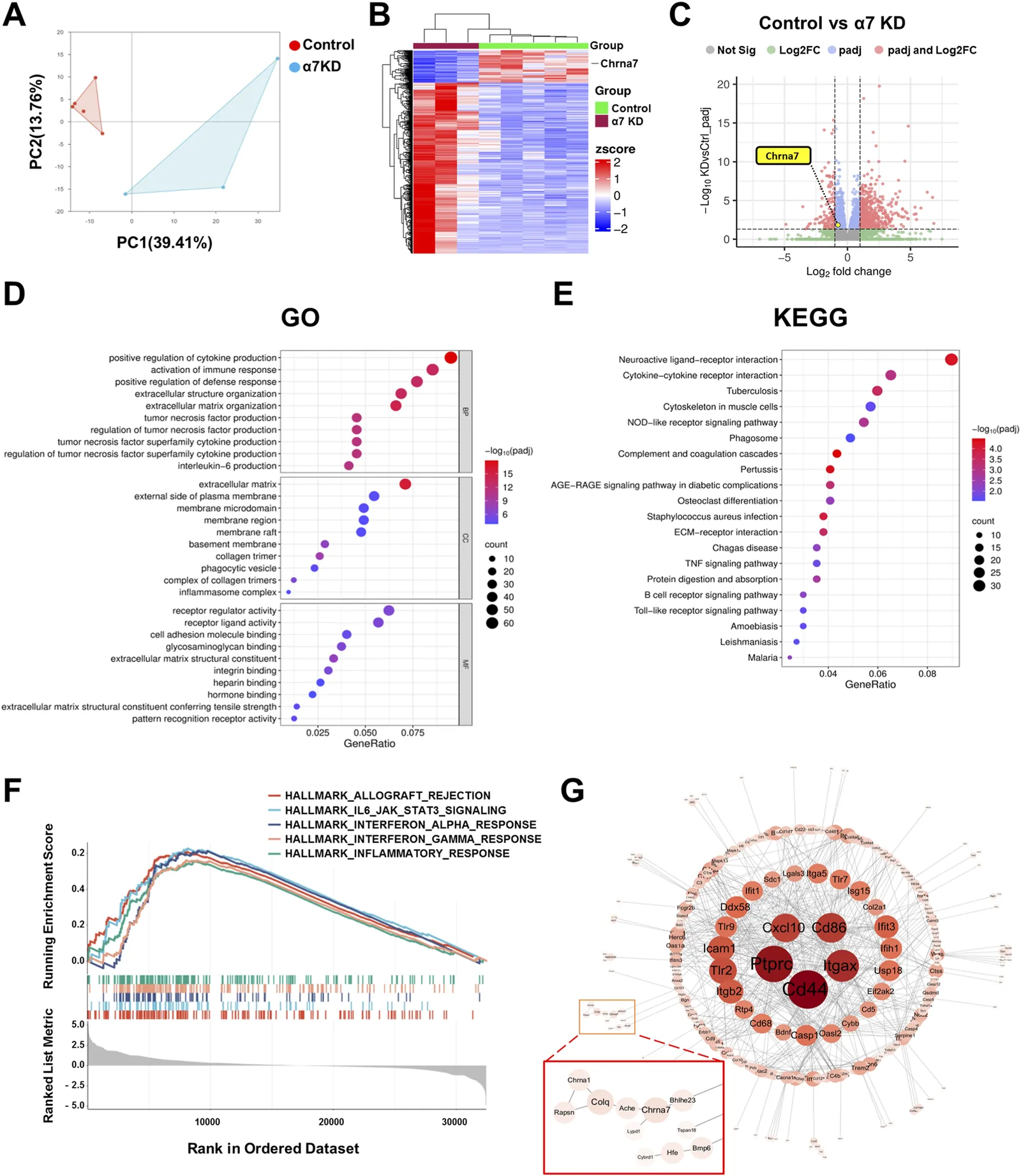
Transcriptomic analysis in α7 KD mice. *n* = 5 control mice and 3 α7 KD mice. **(A)** Principal component analysis (PCA). **(B)** Cluster analysis of DEGs determined by RNA sequencing of control *versus* α7 KD mice. **(C)** Volcano plot showing DEGs in control *versus* α7 KD mice (|log2fold-change| ≥ 1 and padj <0.05). **(D)** GO enrichment analysis of DEGs. **(E)** KEGG pathway enrichment analysis of DEGs. **(F)** Top-five hallmark genesets sorted by normalized enrichment score (NES) comparing control *versus* α7 KD mice. **(G)** The PPI network of overall DEGs, determined by STRING in high confidence (The minimum required interaction score was set as 0.7). From the inside out, genes are categorized and displayed based on their degree of connectivity: five genes with a degree greater than 20, 23 genes with a degree between 11 and 20, and remaining genes with a degree less than or equal to 10.

To further detail how α7 nAChR knockdown affects the hippocampus, we sequenced RNA acquired from the hippocampus to analyze transcriptome-wide changes that arise from the decrease of α7 nAChR. Principal component analysis (PCA) of our RNA sequencing data revealed that α7 KD and control samples clustered at opposite sides of the PC1 axis, and PC2 axis scattering was more pronounced for α7 KD *versus* control mice (Figure 7A). Hierarchical clustering showed that control mice gene expression patterns were similar between samples, whereas those among α7 KD mice varied considerably. This increased dispersion may be attributed to variations in the infection efficiency of rAAV within the hippocampus of the mice. Despite this, the expression profiles were significantly different between control and α7 KD mice (Figure 7B). Compared to control mice, 866 transcripts were differentially expressed in α7 KD mice, among which 729 genes were upregulated and 137 genes were downregulated (Figure 7C). To identify biological processes discernably affected, we analyzed the obtained DEGs using GO enrichment analysis. As shown in Figure 7D, the DEGs were mainly enriched in GO terms related to inflammatory responses and extracellular matrix (ECM), such as positive regulation of cytokine production, activation of immune response and ECM organization. To further access the underlying signaling pathways, we performed KEGG analysis on the DEGs. The results showed the DEGs were mainly enriched in KEGG terms related to signal transduction and immunity, such as neuroactive ligand-receptor interaction, cytokine-cytokine receptor interaction and NOD-like receptor signaling pathway (Figure 7E). To identify distinctive transcriptional programs activated by α7 nAChR knockdown, a GSEA analysis was performed using 50 hallmark gene sets collection from MSigDB. Noticeably, the top five sets enriched in α7 KD mice were linked to immune and inflammation pathways (Figure 7F). Taken together, these findings suggest that the knockdown of α7 nAChR in the hippocampus results in an excessive activation of the immune response, remodeling of the neural microenvironment, and abnormalities in neuronal signal transduction.

To identify DEGs with significant regulatory roles, we utilized Cytoscape to conduct an analysis of the PPI network. The five genes exhibiting the highest degree of connectivity were identified as *Cd44*, *Ptprc*, *Itgax*, *Cd86*, and *Cxcl10* (Figure 7G). These genes all exhibited upregulation in α7 KD mice. Additionally, we emphasize the sub-network containing the specifically knocked-down gene *Chrna7*, which encompasses the upregulated genes *Ache*, *Colq*, *Rapsn*, and *Chrna1*, as well as the downregulated genes *Bhlhe23* and *Lypd1*.

### 3.8 Knocking out α7 nAChR with another rAAV also caused AD-like pathology

**FIGURE 8:**
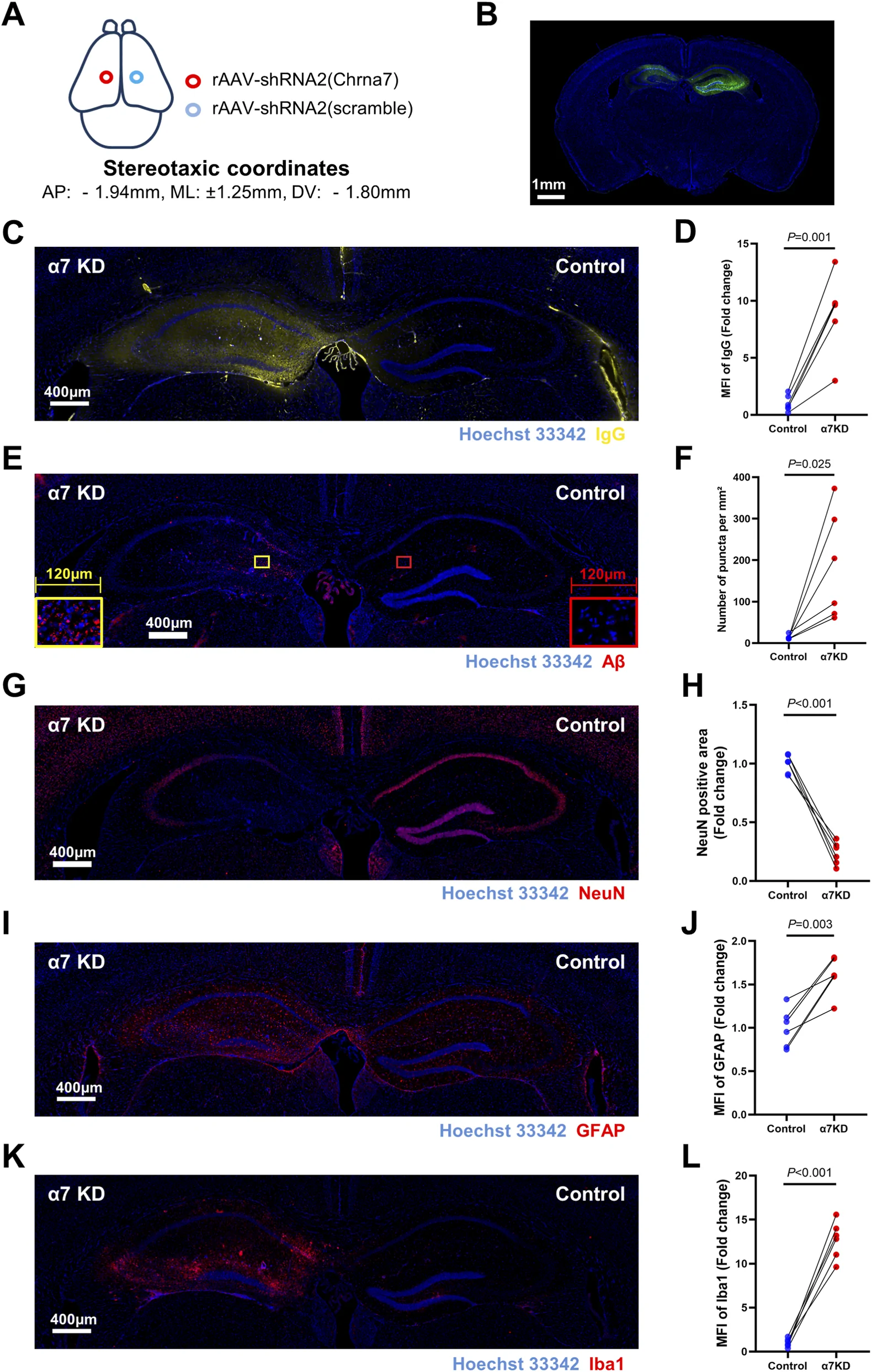
The application of a second rAAV to knockdown α7 nAChR induced AD-like pathology. The left hippocampus received α7 nAChR knockdown rAAV, while the right received control rAAV. *n* = 6 mice per group. **(A)** Schematic of the second rAAV injection in 4-month-old mice. **(B)** Representative EGFP fluorescence images showing the injection sites of rAAV in mouse hippocampus. **(C)** Representative images of IgG immunofluorescence staining. **(D)** The MFI of IgG in hippocampus (*t* = 7.395, df = 5). **(E)** Representative images of Aβ immunofluorescence staining. **(F)** Quantitative analysis of Aβ burden in hippocampus (*t* = 4.598, df = 5). **(G)** Representative images of NeuN immunofluorescence staining. **(H)** Quantification of the NeuN positive area in hippocampus (*t* = 27.80, df = 5). **(I)** Representative images of GFAP immunofluorescence staining. **(J)** The MFI of GFAP in hippocampus (*t* = 8.390, df = 5). **(K)** Representative images of Iba1 immunofluorescence staining. **(L)** The MFI of Iba1 in hippocampus (*t* = 11.81, df = 5). Lines connect control and α7 KD data points of the same mouse. *P* values were determined by paired Student’s t-.test.

To exclude potential off-target effects, a second rAAV was employed to specifically knock down the α7 nAChR. One month post-injection, the pathology associated with AD was assessed. The rAAV targeting α7 nAChR knockdown was administered into the left hippocampus of the mice, while the control rAAV was injected into the right hippocampus (Figure 8A). The injection sites were verified by fluorescence imaging (Figure 8B). In concordance with the above results of the first rAAV, the application of the second rAAV to suppress α7 nAChR expression also elevated total Aβ burden (*P* = 0.025) and endogenous IgG levels (*P* = 0.001), increased the MFI of GFAP (*P* = 0.003) and Iba1 (*P* < 0.001), and reduced the area of NeuN-positive neurons (*P* < 0.001) (Figures 8C–L). However, we did not observe the aggregation of p-Tau (Thr181) (data not shown).

## 4 Discussion

Given the longstanding debate surrounding the role of α7 nAChR in AD, it is crucial to elucidate the consequences of α7 nAChR loss during adulthood. Our findings provide novel evidence that a reduction in α7 nAChR levels within the hippocampus of mice at 4 months of age rapidly induced AD-like phenotypes. Notably, abnormal behavior was observed 1–2 months following rAAV injection, whereas increases in Aβ levels, p-Tau aggregation, glial cell activation, and extensive changes in gene expression were detected 2 months post-injection. The accuracy and reliability of these results are enhanced by the avoidance of systematic and non-specific interference in the gene knockout model. Furthermore, the emergence of these AD-like pathologies occurs significantly faster than previously reported in α7 nAChR knockout mice (1–2 months *versus* 12 months) (Tropea et al., 2021), potentially due to our method circumventing the potential developmental compensation issues in α7 nAChR knockout mice. These findings substantiate the notion that the loss of α7 nAChR in the hippocampus of adult individuals constitutes a significant risk factor for AD, underscoring α7 nAChR as a promising therapeutic target for AD intervention.

AD patients often suffer from progressive memory loss and severe cognitive dysfunction, accompanied by neuropsychiatric symptoms and behavior changes (Chakraborty et al., 2019). As a core limbic brain region, the hippocampus serves a central role in governing cognitive processing, attentional regulation, and the encoding and consolidation of memory (Letsinger et al., 2022). Given the deficiencies of hippocampal α7 nAChR in AD patients and its important role in cognition (Egea et al., 2015; Hellström-Lindahl et al., 1999; Leiser et al., 2009), and the significant pro-cognitive effects exhibited by α7 nAChR agonists such as cotinine in both Alzheimer’s disease and aged murine models (Grizzell et al., 2017; Sadigh-Eteghad et al., 2020), it is plausible to hypothesize that behavioral and cognitive changes in AD are associated with the loss of α7 nAChR. Our results showed that the α7 KD mice displayed significant cognitive dysfunction, consistent with previous reports in AD patients (Chakraborty et al., 2019). In addition, α7 KD mice also showed excessive motor activity in the open field, suggesting the presence of agitation. However, there was no significant alteration in the time spent by α7 KD mice in the center of the open field, indicating an absence of anxiety. It is noteworthy that both anxiety and agitation are prevalent clinical symptoms of AD, with anxiety typically manifesting in the early stages and often being supplanted by agitation as the disease progresses (Koenig et al., 2016; Mendez, 2021; Ruthirakuhan et al., 2018). Evidence suggests that agitation affects 20%–50% of individuals with moderate to severe AD (Ruthirakuhan et al., 2018). Consequently, the reduction in α7 nAChR levels may be indicative of more advanced AD progression. In hippocampal neurons, α7 nAChR activation drives robust intracellular calcium influx and downstream calcium-dependent signaling cascades, which potentiates presynaptic glutamate release and sustains glutamatergic synaptic plasticity—an essential cellular foundation for memory formation (Cheng and Yakel, 2015). Therefore, diminished α7 nAChR abundance directly impairs hippocampal synaptic plasticity, ultimately resulting in severe deficits in spatial reference and working memory. Furthermore, elevated Aβ deposition and glial hyperactivation were detected in our α7 KD mice. Nevertheless, whether these pathological events act as intermediate mediators linking α7 nAChR loss to cognitive decline remains to be fully elucidated in follow-up investigations.

The accumulation of cerebral Aβ is a neuropathological hallmark of AD (Reiss et al., 2018). Prior research has demonstrated an elevated level of Aβ in α7 knockout mice at 12 months of age (Tropea et al., 2021). In our study, we similarly observed Aβ accumulation in the hippocampus following a 2-month knockdown of α7 nAChR, which was consistent with previous studies based on α7 knockout mice. In addition, α7 nAChR agonist cotinine can attenuate cerebral Aβ deposition and block Aβ oligomerization in AD mouse models, further supporting the involvement of α7 nAChRs in the regulation of Aβ pathology (Echeverria et al., 2011). Previous studies suggest that the knockdown of α7 nAChR may lead to an increase in Aβ levels through several mechanisms. Firstly, α7 nAChR plays a role in the regulation of APP processing, promoting a shift towards non-amyloidogenic pathways, thereby reducing Aβ production (Mousavi and Hellström-Lindahl, 2009; Nie et al., 2010). Consequently, a reduction in α7 nAChR expression may disrupt normal APP processing and result in excessive Aβ production. Secondly, Aβ is known to bind to α7 nAChR, potentially serving a physiological function (Gulisano et al., 2019; Puzzo et al., 2015). Thus, it is hypothesized that a decrease in α7 nAChR expression may trigger a compensatory increase in Aβ levels. Lastly, the binding of Aβ to α7 nAChR facilitates the endocytosis of the Aβ-α7 nAChR complex by neurons and astrocytes (Fontana et al., 2023). Therefore, a reduction in α7 nAChR may impair Aβ endocytosis, leading to its extracellular accumulation. In the present work, the accumulation of Aβ arises primarily from upregulated APP expression instead of modified Aβ processing process, given that BACE1 expression remained unaltered across groups. Notably, unlike human APP-transgenic mouse models, Aβ pathology in α7 KD mice manifests as granular aggregates rather than large amyloid plaques. This discrepancy may be explained by the fact that endogenous mouse Aβ has a weaker propensity to form mature plaques compared with human Aβ (Jankowsky et al., 2007).

The aggregation of Tau proteins represents a fundamental characteristic of AD, and abnormal phosphorylation of Tau is a critical event that initiates tau aggregation within the brains of individuals with AD (Mamun et al., 2020). The phosphorylation site at Tau residue 181 (p-Tau 181) has been directly implicated in AD and is observed to be elevated in the cerebrospinal fluid and blood of AD patients (Hanger et al., 2009; Morrison et al., 2022; Schönknecht et al., 2003). Our findings indicate that the levels of p-Tau 181 in the hippocampus of α7 KD mice did not exhibit an increase; rather, a significant decline was observed. This phenomenon may be attributed to neuronal loss in α7 KD mice. Despite this, we observed a significant increase in the number of p-tau 181 aggregates. In line with our findings showing increased p-Tau deposition in α7 KD mice, existing evidence demonstrates that the α7 nAChR agonist cotinine effectively reduces pathological tau phosphorylation in AD mouse models (Grizzell et al., 2017). Notably, these aggregates were mainly localized in the CA1 region, which is particularly susceptible to the pathology of Tau protein in AD (Lace et al., 2009). The mechanism through which α7 deficiency leads to tau hyperphosphorylation may involve diminished phosphorylation of GSK-3β at the Serine nine residue. This impaired inhibitory modification at Ser 9 results in pathological overactivation of GSK-3β, subsequently causing tau hyperphosphorylation (Tropea et al., 2021).

The loss of neurons is a major pathological feature of AD (Bobinski et al., 1997; Kril et al., 2002). In the hippocampus of α7 KD mice, we observed significant neuronal death, surpassing that observed in α7 knockout mice in prior studies. Notably, due to the mutation of the Cdk3 gene in common lab mouse strains, substantial neuronal death is absent in the AD mouse model (Zhuang et al., 2026). In this context, the serious loss of neurons in the hippocampus of α7 KD mice highlights the severe implications associated with the deletion of α7 nAChR. Under physiological conditions, activation of α7 nAChR exerts diverse effects on hippocampal circuits through various mechanisms. For example, stimulation of α7 nAChRs leads to direct neuronal depolarization and modulation of both glutamatergic and γ-aminobutyric acid (GABA) synaptic transmission (Letsinger et al., 2022). These findings indicate that, in addition to the neurotoxic effects of Aβ and tau pathology, the diminished neuroprotective and neurotrophic functions mediated by α7 nAChR represent an additional mechanism contributing to neuronal death in the hippocampus of α7 KD mice. It is worth noting that no obvious TUNEL-positive signals were detected 2 months after α7 nAChR knockdown, implying that neuronal death might occur at an earlier stage rather than during the late observation period. Therefore, additional detection at earlier time points is required to track the dynamic process of neuronal death.

Neuroinflammation is a principal hallmark of AD, predominantly mediated by the activation of astrocytes and microglia (Kaur et al., 2019; Thakur et al., 2023). Our results showed that the expression of GFAP (an astrocyte marker) and Iba1 (a microglia cell marker) was increased in the hippocampus of α7 KD mice, indicating the activation of astrocytes and microglia. Consistent with these histological observations, transcriptomic profiling also uncovered prominent enrichment of inflammation-related signaling cascades in the α7 KD hippocampus. Immunofluorescence staining with goat anti-mouse IgG fluorescent antibody also showed that IgG level increased, which represented that the blood-brain barrier was damaged, which may be related to the continuous occurrence of inflammation. The damage of blood-brain barrier will recruit more immune cells and cytokines into brain parenchyma, and then induce neurodegeneration (Chen et al., 2023). Moreover, five hub genes, *Cd44*, *Ptprc*, *Itgax*, *Cd86*, and *Cxcl10*, identified by PPI analysis, are all implicated in immune regulation and have been reported to be associated with AD (Bradburn et al., 2018; Huang et al., 2024; Li and De Muynck, 2021; Liu et al., 2022; Pinner et al., 2017). Notably, the α7 nAChR in astrocytes and microglia plays a crucial role in mediating anti-inflammatory responses. The anti-inflammatory effects mediated by α7 nAChRs rely on Jak2 activation. Jak2 activation blocks p65 nuclear entry to reduce ROS and cytokine release, while concurrently triggering PI3K/Akt signaling to drive Nrf2 nuclear translocation and HO-1 overexpression, thereby conferring potent anti-inflammatory and neuroprotective effects (Egea et al., 2015). In addition, Aβ is known to trigger inflammatory responses (Dorey et al., 2014). Therefore, the downregulation of α7 nAChR may precipitate neuroinflammation by concurrently diminishing anti-inflammatory mechanisms and elevating Aβ levels. Sustained neuroinflammation drives excessive release of pro-inflammatory cytokines and mediators, neuronal degeneration, impaired astrocytic glutamate uptake, synaptic loss, and ultimately cognitive impairment characteristic of AD (Kaur et al., 2019). Notably, glial activation exerts dual stage-dependent functions during AD progression. Moderately activated microglia and astrocytes confer compensatory neuroprotection by facilitating cerebral Aβ clearance (Fakhoury, 2018). Consistent with this protective property, we did not detect widespread upregulation of inflammatory cytokines at the 2-month observation time point. Such modest changes in cytokine abundance seem contradictory to our transcriptomic data, which revealed prominent enrichment of inflammatory signaling cascades in α7 KD hippocampus. Given the absence of serial sampling at early time points after α7 nAChR knockdown, the intensity of early inflammatory responses cannot be confirmed definitively. It remains unclear whether acute, robust neuroinflammation occurs in the initial stage, even though only mild selective cytokine changes are observed at the 2-month endpoint.

Transcriptomic analysis also indicates a disruption in the ECM within the hippocampus of α7 KD mice. The ECM is a structural network composed of various macromolecules secreted by cells, and plays a critical role in numerous aspects of AD pathology. Disruption of the ECM affects synaptic transmission, Aβ pathology, Tau pathology, oxidative stress, and inflammatory responses (Sun et al., 2021). Consequently, ECM may play a role in AD-like pathologies associated with the downregulation of α7 nAChR.

In the sub-network encompassing the knocked-down gene *Chrna7*, alterations were observed in the expression of genes associated with cholinergic transmission. The *Ache* gene encodes acetylcholinesterase (AChE), an enzyme responsible for the degradation of acetylcholine (Dvir et al., 2010), while the collagen-like tail subunit encoded by the *Colq* gene enhances the localization and stability of AChE at the postsynaptic membrane (Nakata et al., 2013). Notably, the knockdown of the α7 nAChR resulted in the upregulation of both *Ache* and *Colq* gene expression, which may contribute to a reduction in acetylcholine levels observed in AD. The expression product of *Lypd1* serves as a negative regulator of α7 nAChR activity (Anderson et al., 2025). The *Rapsn* gene encodes a postsynaptic protein essential for the aggregation of nicotinic acetylcholine receptors (Ferns, 2021), while the *Chrna1* gene encodes the cholinergic receptor nicotinic α1 subunit. Knockdown of α7 nAChR resulted in a reduction of *Lypd1* expression and an upregulation of *Rapsn* and *Chrna1* expression, suggesting a compensatory mechanism in response to the diminished levels of α7 nAChR. The *Bhlhe23* gene product may modulate the expression of genes required for the differentiation and/or maintenance of neuronal cell (Bramblett et al., 2002), a reduction in *Bhlhe23* expression could be a critical mechanism underlying neuronal death associated with α7 nAChR knockdown.

In the hippocampus of AD patients, the overall abundance of α7 nAChR declines substantially. However, cell-subtype-specific alterations can be distinguished: α7 nAChR expression is suppressed in neurons, while reactive astrocytes surrounding neuritic plaques display elevated α7 nAChR expression, a phenomenon likely triggered by sustained interactions with Aβ species (Fontana et al., 2023). Under physiological conditions, neuronal α7 nAChRs regulate the release of neurotransmitters such as glutamate and GABA. Neuron-released GABA facilitates astrocytic secretion of the gliotransmitter ATP, which retrogradely restrains glutamate release from neurons (Rennie, 2025). Meanwhile, activation of astrocytic α7 nAChRs triggers the release of multiple gliotransmitters including ATP, D-serine, GABA and glutamate, which directly modulate neuronal function (Fontana et al., 2023). Accordingly, this cell-type-specific shift in α7 nAChR abundance implies that α7 nAChR deficiency disrupts physiological neuron–astrocyte crosstalk. In addition, tau pathology triggers Mef2c/miR-133a/A1R signal and drives excessive neuronal secretion of Lcn2, which acts as a critical mediator governing neuron-glia crosstalk. Lcn2 triggers aberrant activation of both astrocytes and microglia, subsequently provoking neuroinflammation and synaptic deterioration in neurons (Zhou et al., 2023). Collectively, α7 nAChR deficiency may disrupt physiological neuron–astrocyte crosstalk *via* impaired neurotransmitter homeostasis and progressive tau pathology, thereby potentially exacerbating the progression of AD pathology.

Furthermore, cyclic estrogen fluctuations in female mice lead to significant variations in hippocampal α7 nAChR levels and neuroinflammatory phenotypes (Wang et al., 2023). Therefore, our study exclusively utilized male mice to mitigate the impact of estrogen fluctuations on the consistency of shRNA-mediated α7 nAChR knockdown outcomes. This represents the limitations of this study, and including both male and female animals will be an important goal in future research to understand whether gender-related factors (such as estrogen) affect the consequences of the decrease of α7 nAChR level.

In light of the pathological characteristics of AD, previous research has proposed various hypotheses, including those centered on Aβ, Tau proteins, cholinergic dysfunction, and neuroinflammation (Chen et al., 2022; Thakur et al., 2023). However, the predominant mechanism remains a subject of debate. Although AD may result from multiple contributing factors, the data presented herein offer compelling evidence that the decrease of hippocampal α7 nAChR level in adulthood is sufficient to induce AD-like phenotypes. Our findings may also elucidate why AChE inhibitors or α7 nAChR agonists are able to alleviate the symptoms of AD but are unable to halt its progression (Ogos et al., 2024). Specifically, AD is characterized by cholinergic neuron dysfunction in the basal forebrain and a deficiency of α7 nAChR in the hippocampus (Giacobini et al., 2022; Hellström-Lindahl et al., 1999). While these pharmacological agents can elevate acetylcholine levels or mimic its effects, they do not address the underlying decline in receptor levels. The absence of α7 nAChR in the hippocampus is at least as critical as the dysfunction of the cholinergic system in the basal forebrain, as both contribute to the impairment of cholinergic transmission. These insights have the potential to inform the development of therapeutic strategies targeting the cholinergic system in AD. Specifically, they suggest a focus on restoring the expression and normal function of the α7 nAChR, rather than merely augmenting acetylcholine levels. A therapeutic approach that concurrently restores both ligand and receptor levels may prove more efficacious in decelerating or ameliorating the progression of the disease in patients.

## 5 Conclusion

In summary, this study elucidates the characteristics of the decrease of α7 nAChR in adult mice hippocampus to enhance the understanding of its role in AD pathology. Knocking down α7 nAChR in the hippocampus of 4-month-old mice can rapidly induce key pathological processes related to AD, including Aβ accumulation, Tau protein aggregation, extensive neuronal damage, and neuroinflammation. Additionally, within 1–2 months following the knockdown, the mice demonstrated cognitive memory impairments and behaviors suggestive of agitation. While previous studies have supported the role of cholinergic system dysfunction in AD, the therapeutic efficacy of increasing acetylcholine levels through cholinesterase inhibitors has been limited. Our findings contribute to a more nuanced understanding of the cholinergic hypothesis by proposing that impairment of cholinergic receptors, particularly the α7 nAChR, may play a pivotal role in AD pathology. Thus, maintaining α7 nAChR levels and function could represent an effective strategy for the treatment and prevention of AD.

## Data Availability

The RNA-sequencing data are available in the GSA database (https://ngdc.cncb.ac.cn/gsa/, CRA038911). The raw data supporting the conclusions of this article will be made available by the authors, without undue reservation.
