# Supplementary material for: Adult-onset hippocampal α7 nicotinic acetylcholine receptor loss rapidly drives Alzheimer-like neuropathology in mice
Source: Front Pharmacol. 2026 Sep 7;17:1929468. doi: 10.3389/fphar.2026.1929468 (PMC13590702; doi:10.3389/fphar.2026.1929468)

Original uncropped membrane of α7 nAChR in Figure 1D:

0.2 s exposure time:

Western blot image

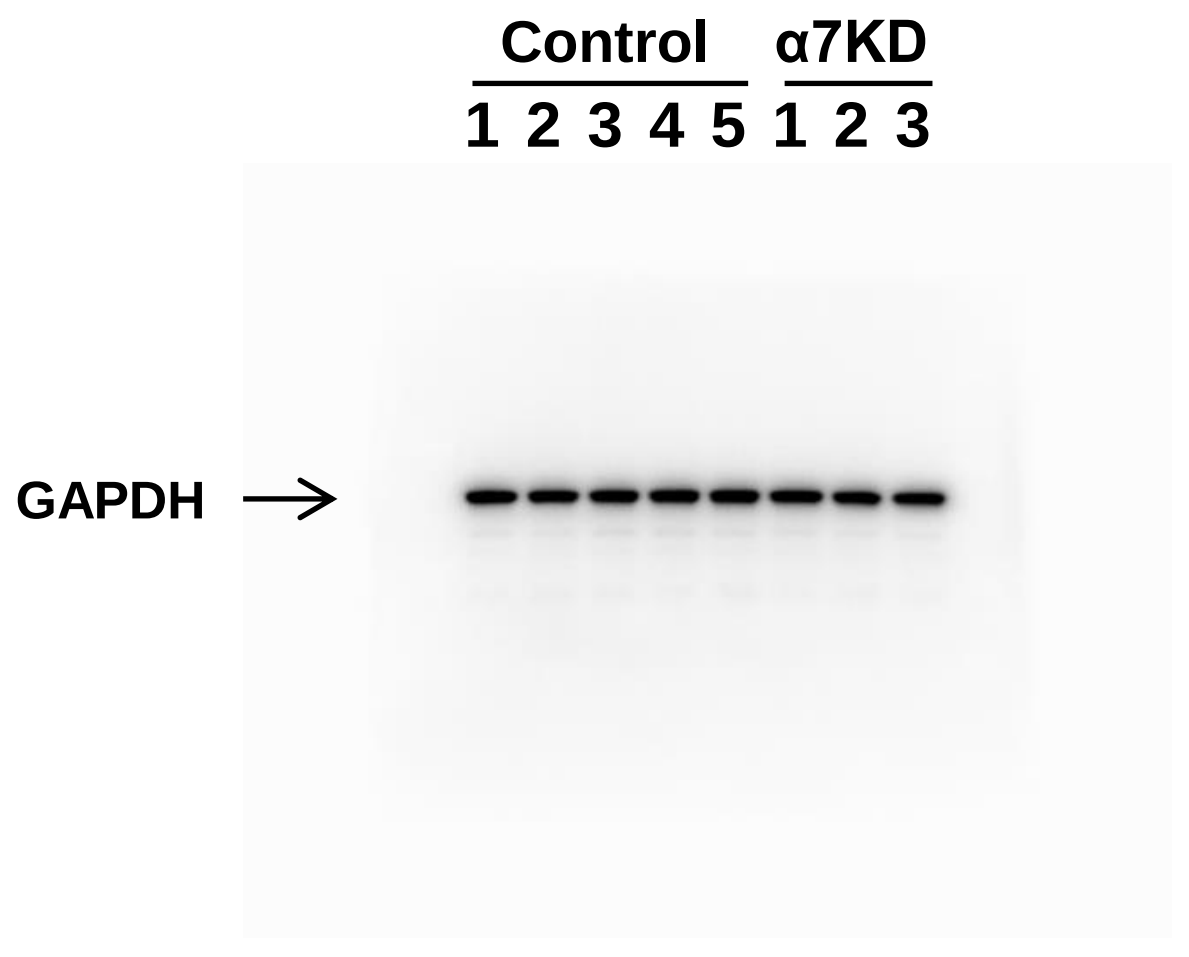

Merge with bright-field

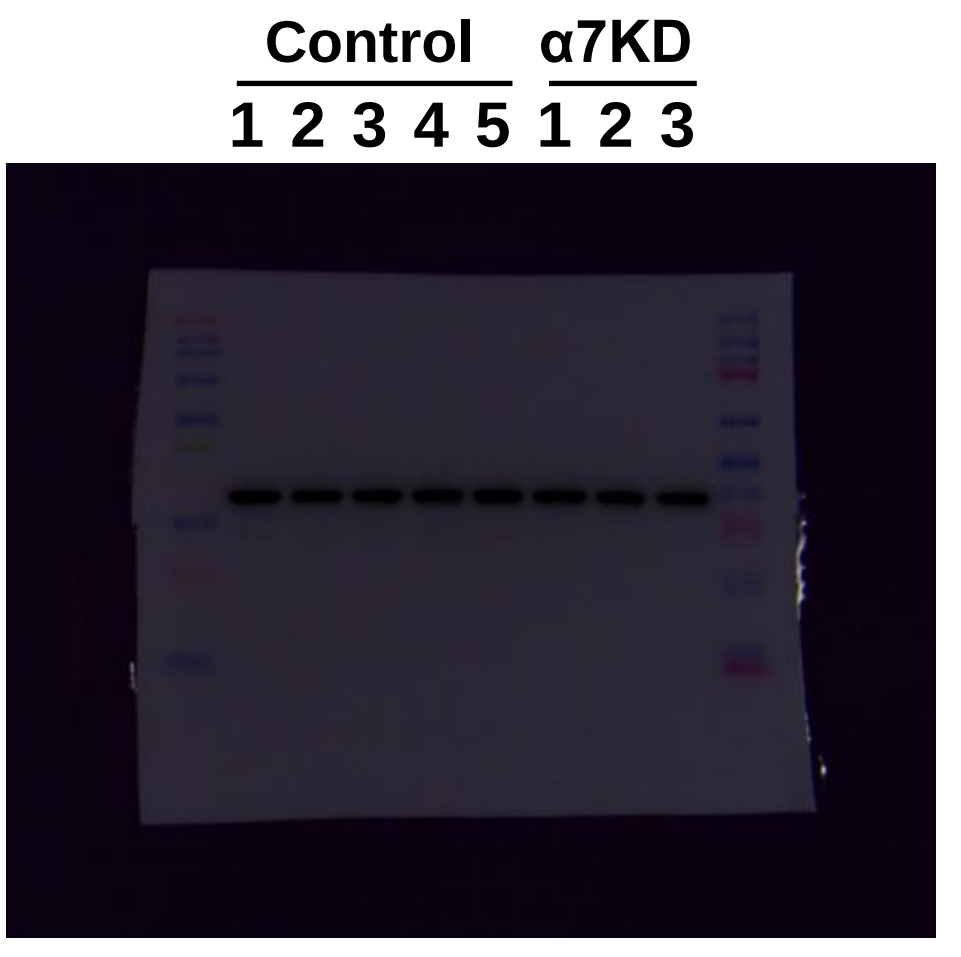

6 s exposure time:

Western blot image

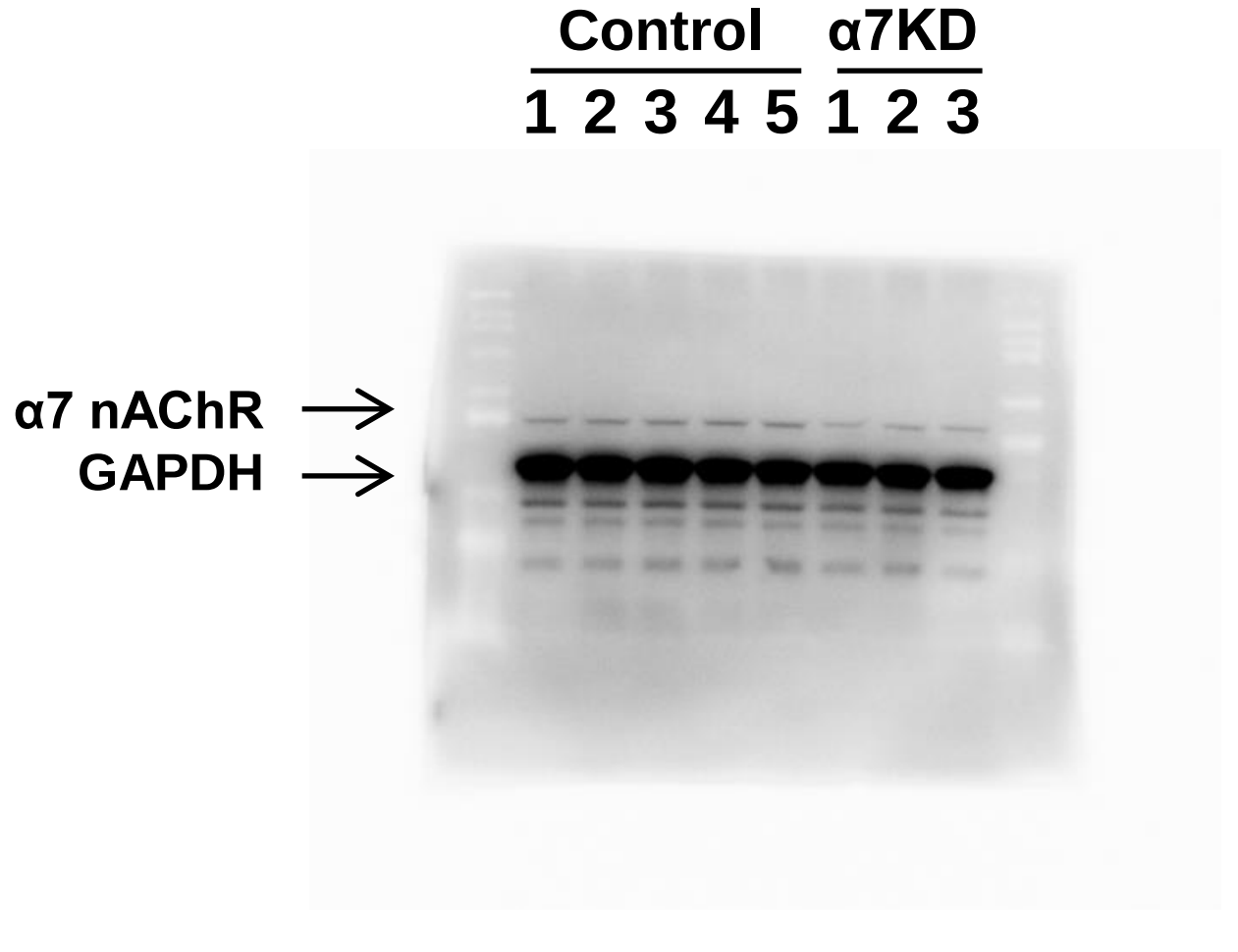

Merge with bright-field

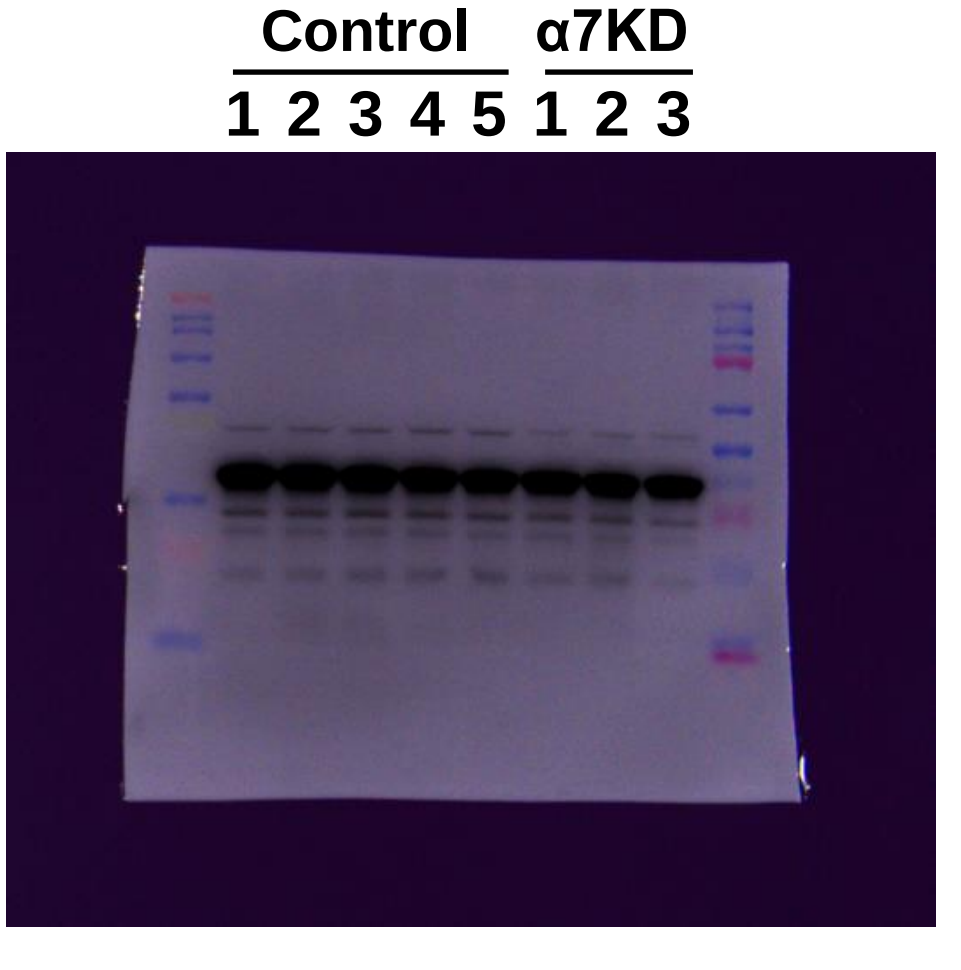

Original uncropped membrane of APP in Figure 3F:

0.5 s exposure time:

Western blot image

Control α7KD
1 2 3 4 5 1 2 3

APP →

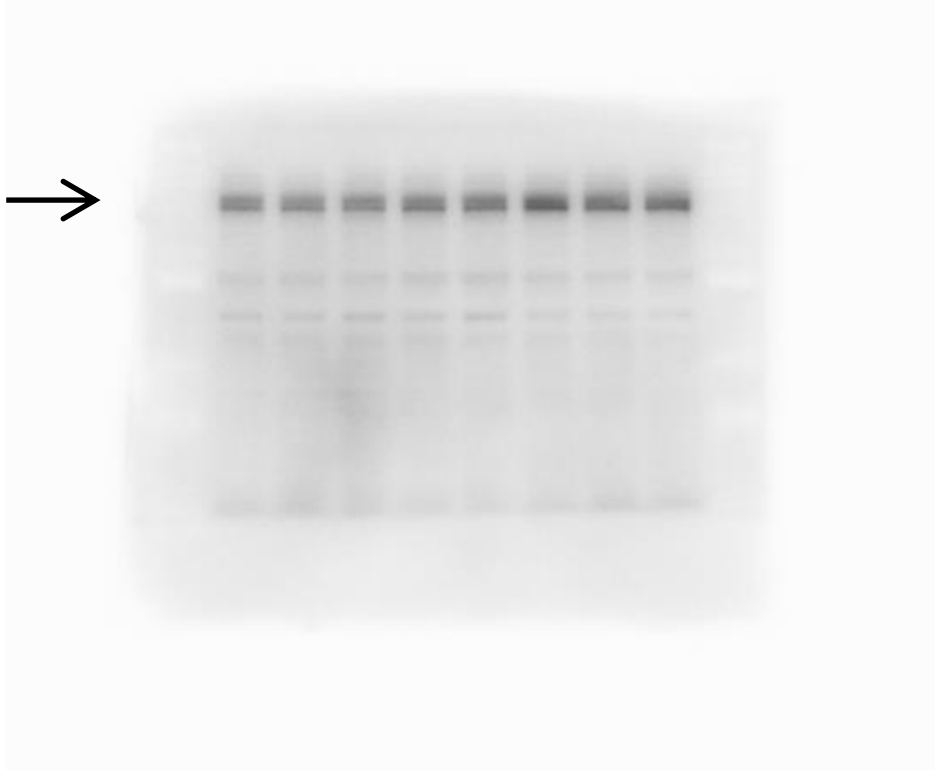

Merge with bright-field

Control α7KD
1 2 3 4 5 1 2 3

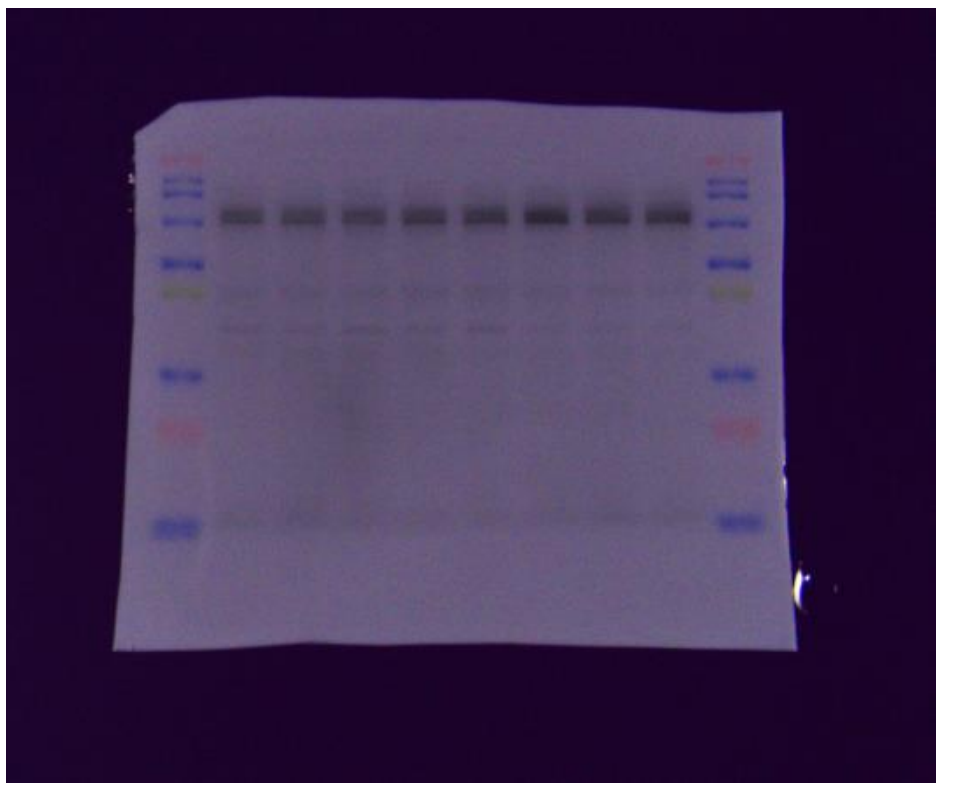

Stripped of APP antibody, the blot was re-probed with GAPDH.

0.2 s exposure time:

Western blot image

Control α7KD
1 2 3 4 5 1 2 3

GAPDH →

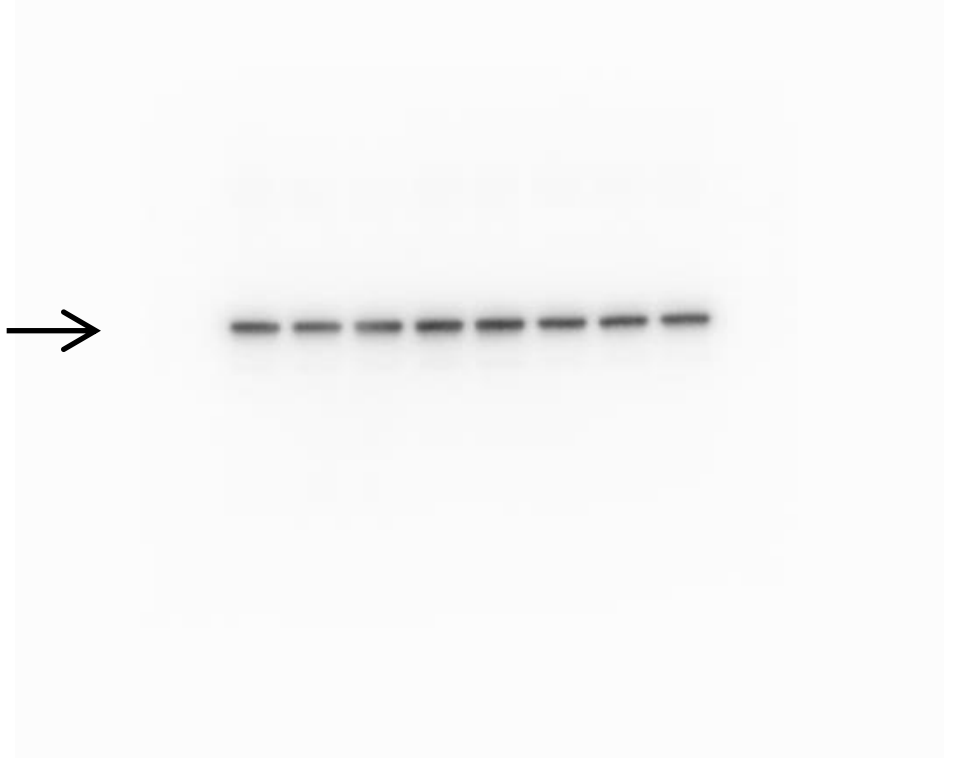

Merge with bright-field

Control α7KD
1 2 3 4 5 1 2 3

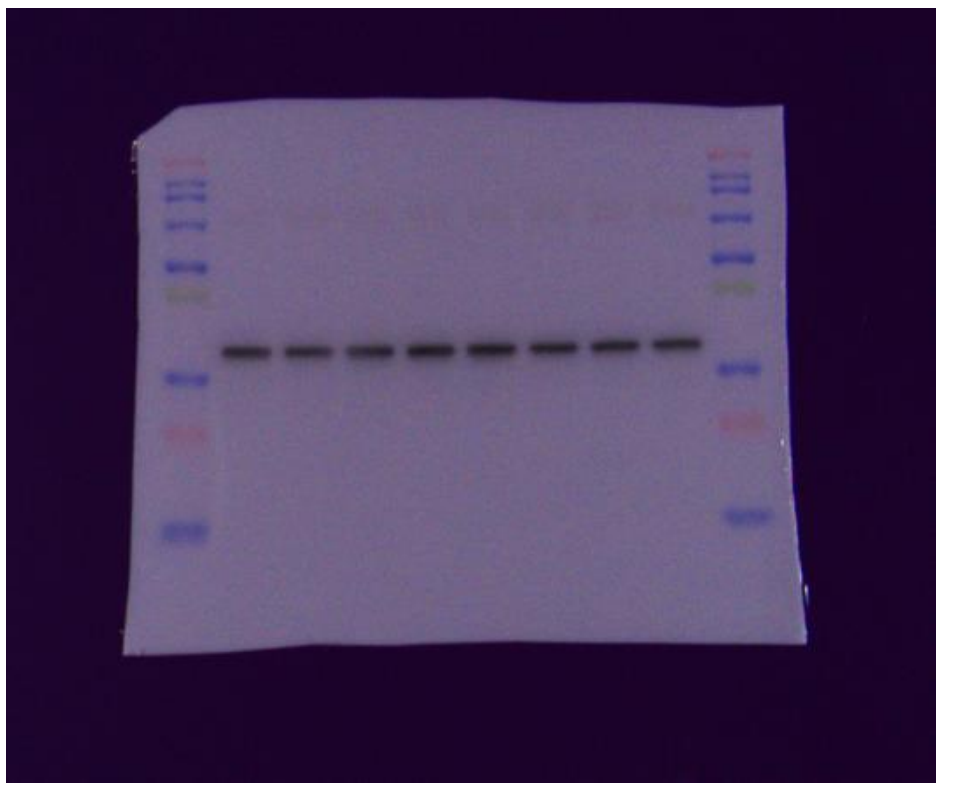

Original uncropped membrane of BACE1 in Figure 3F:

0.8 s exposure time:

Western blot image **Merge with bright-field**

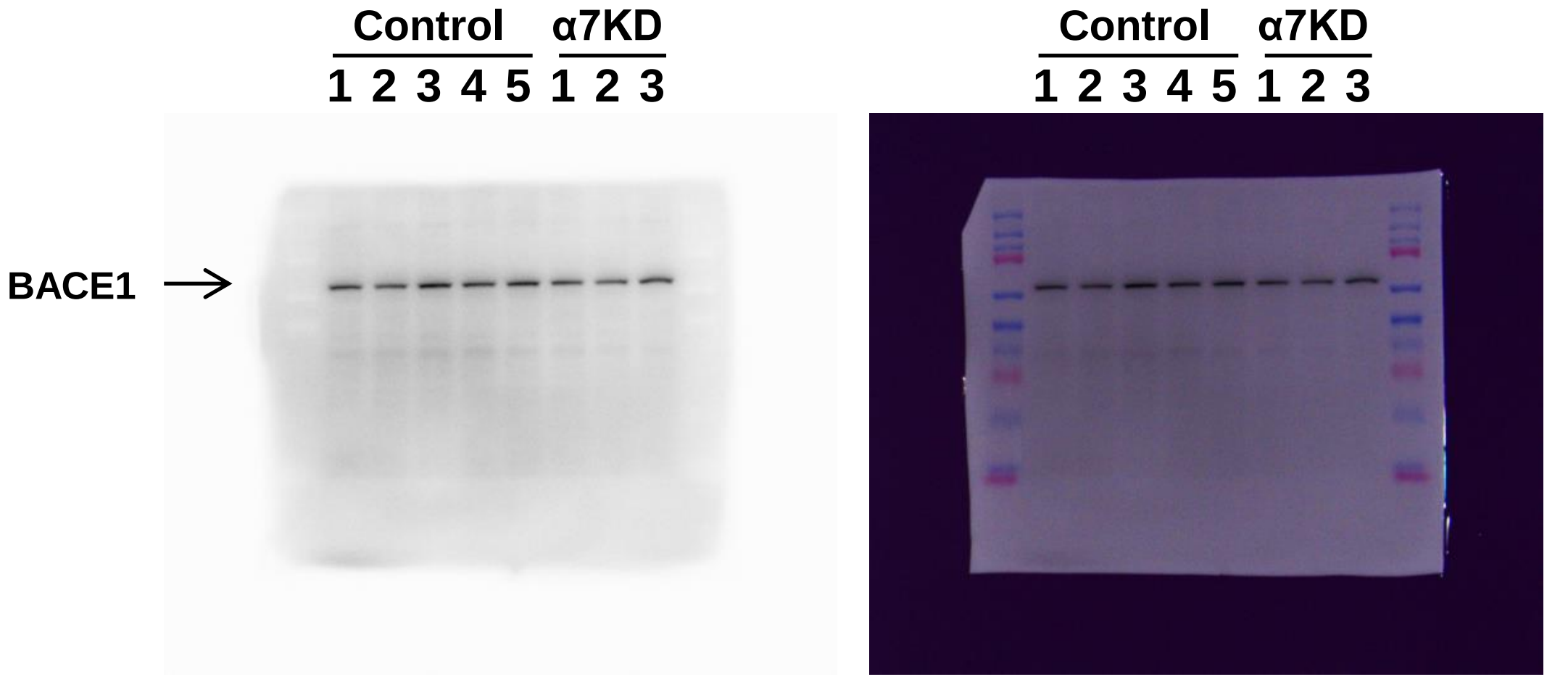

Stripped of BACE1 antibody, the blot was re-probed with GAPDH.

0.1 s exposure time:

Western blot image **Merge with bright-field**

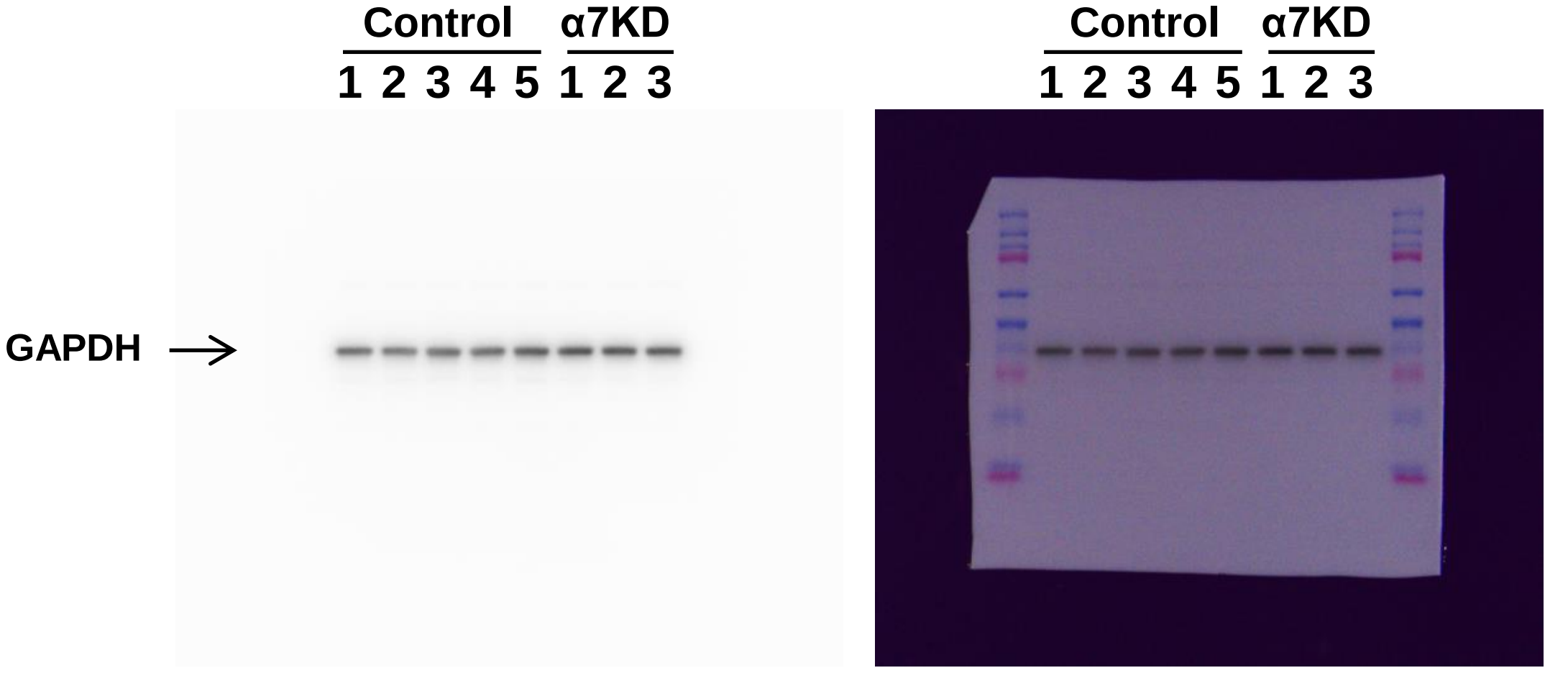

Original uncropped membrane of p-Tau (Thr181) in Figure 4D:

0.2 s exposure time:

Western blot image | **Merge with bright-field**

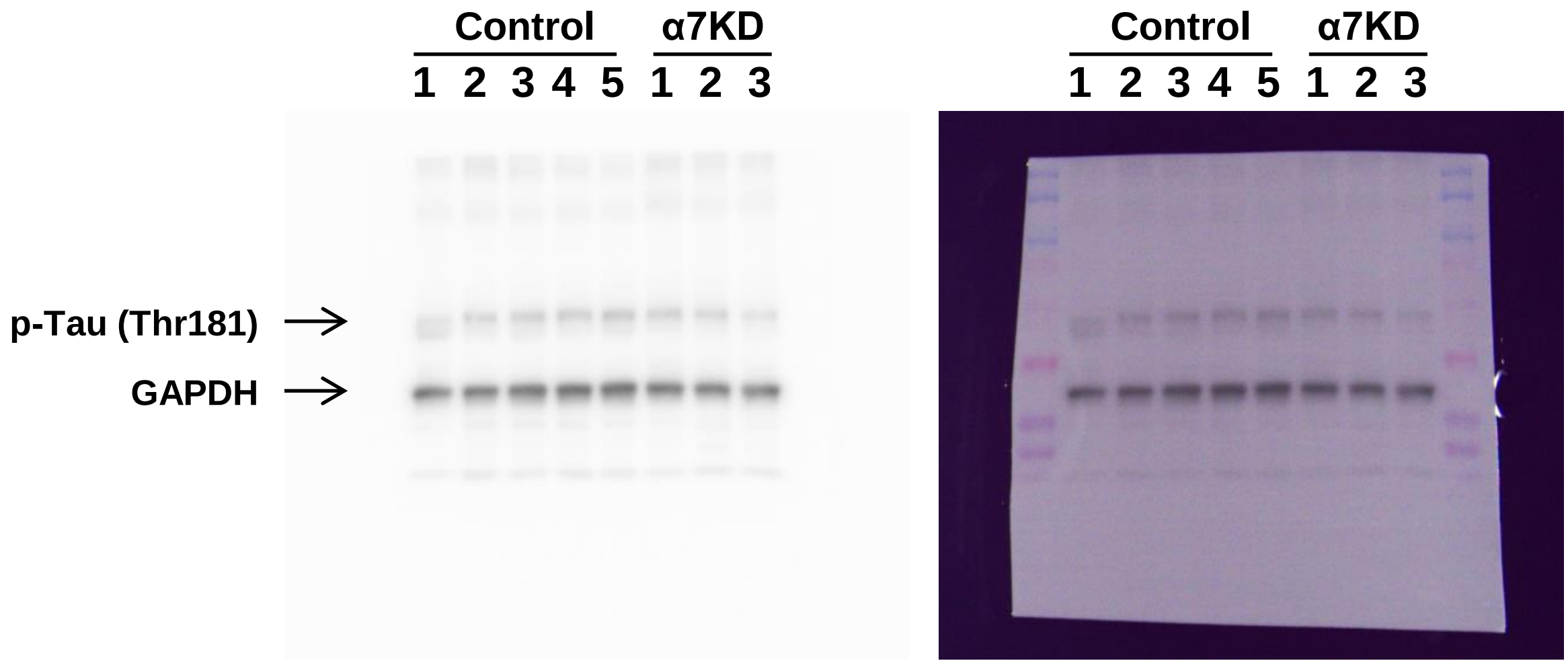

0.4 s exposure time:

Western blot image | **Merge with bright-field**

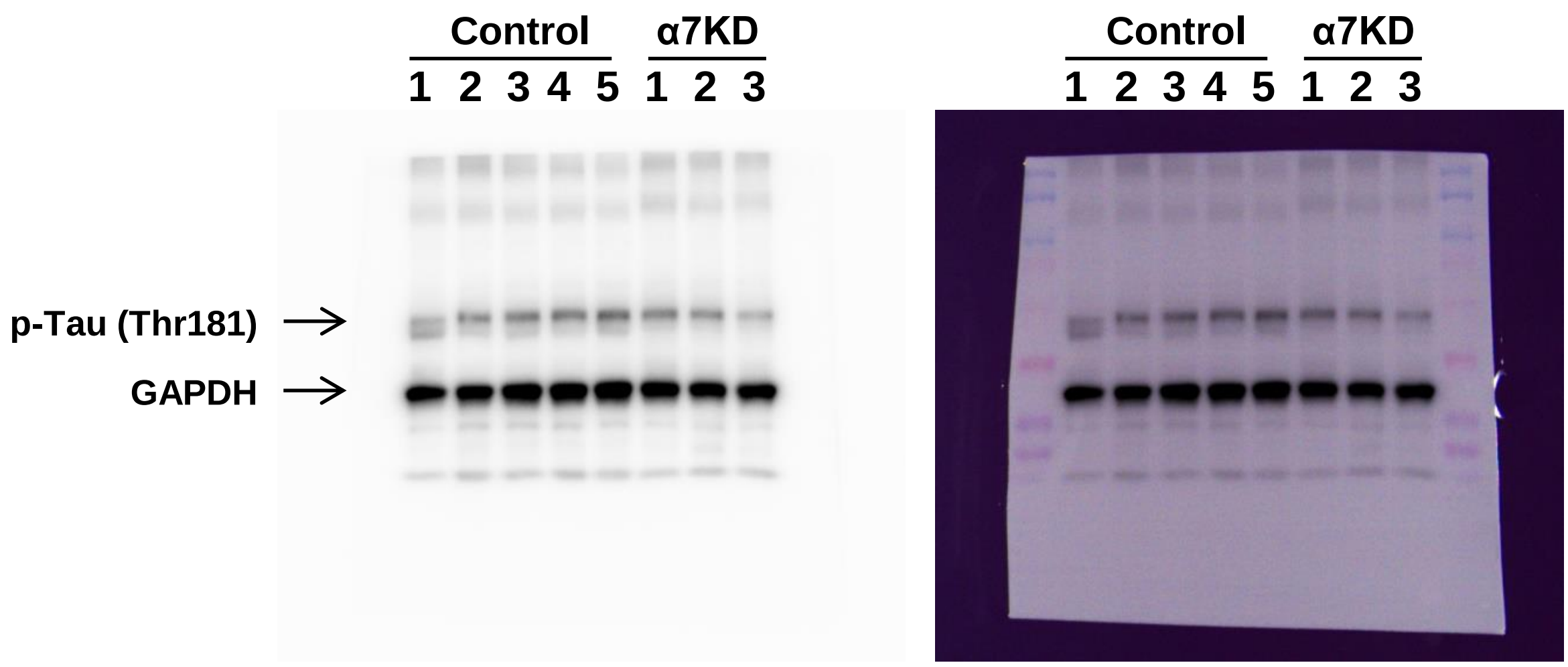

Original uncropped membrane of GFAP in Figure 6E:

0.3 s exposure time:

Western blot image

Control α7KD
1 2 3 4 5 1 2 3

GFAP →
GAPDH →

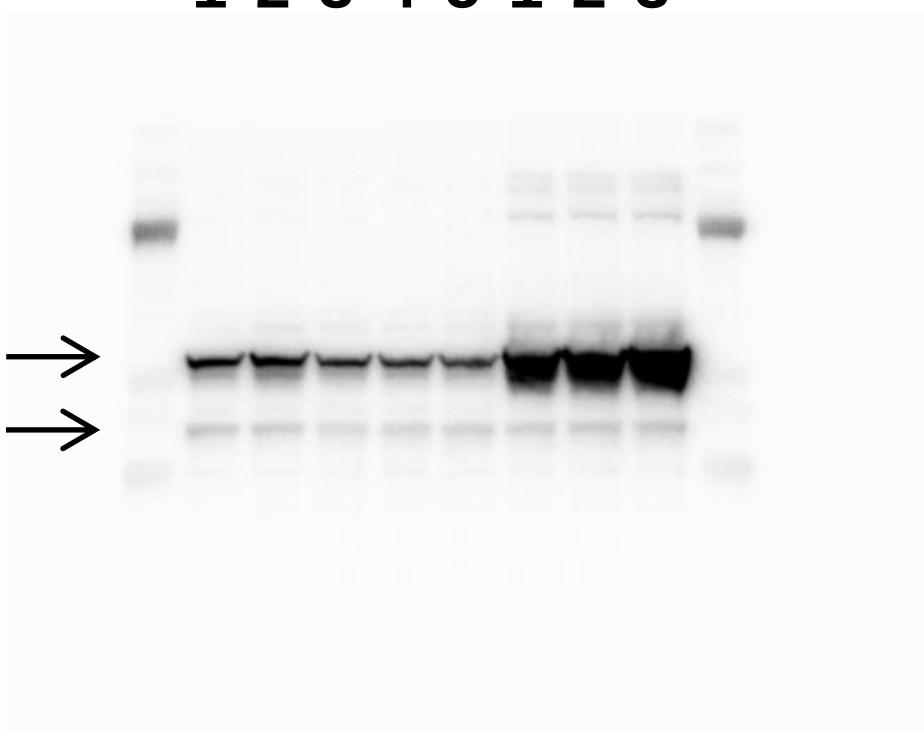

Merge with bright-field

Control α7KD
1 2 3 4 5 1 2 3

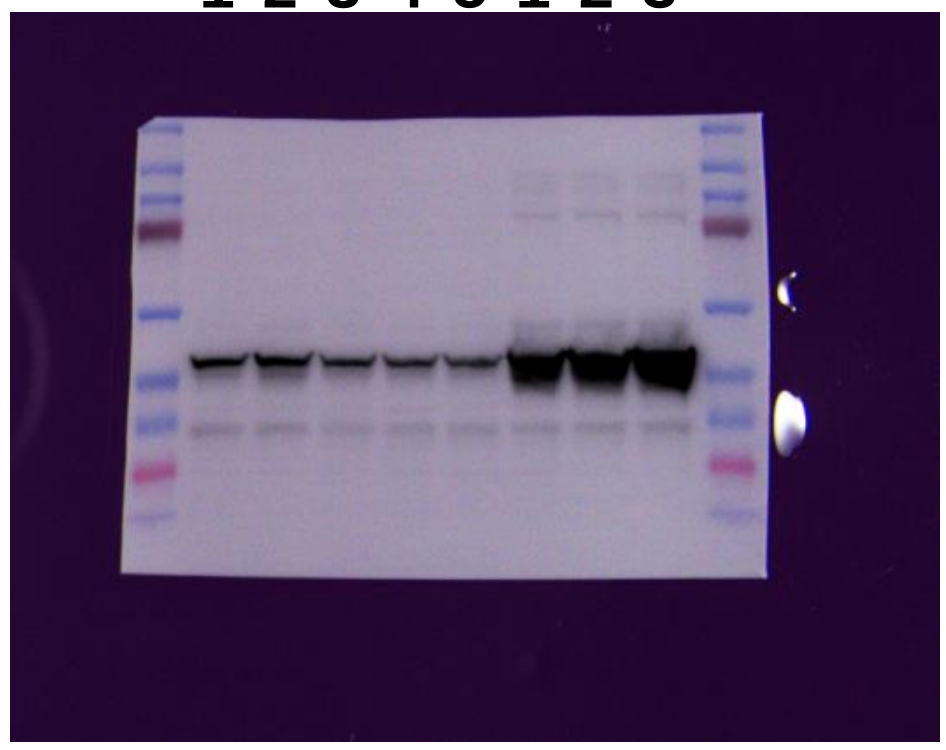

1 s exposure time:

Western blot image

Control α7KD
1 2 3 4 5 1 2 3

GFAP →
GAPDH →

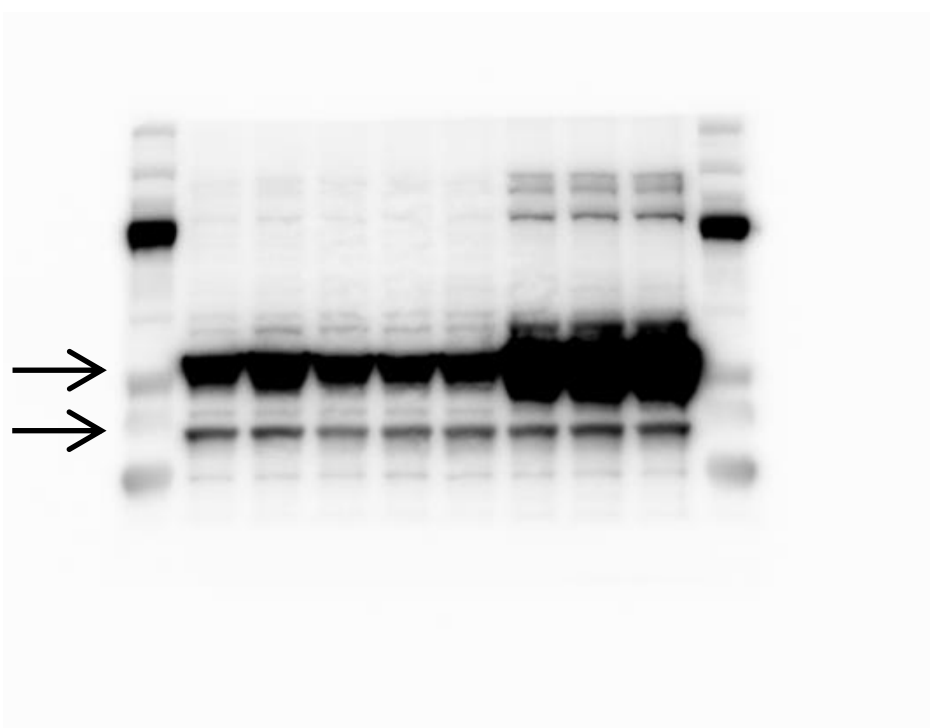

Merge with bright-field

Control α7KD
1 2 3 4 5 1 2 3

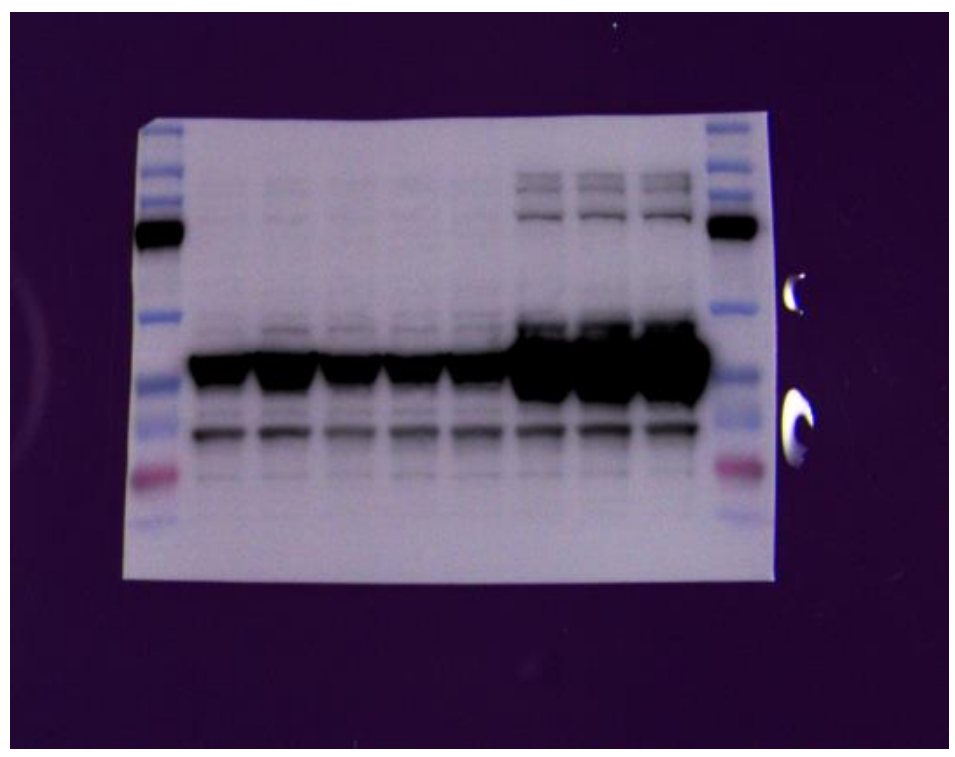

Original uncropped membrane of Iba1 in Figure 6E:

0.3 s exposure time:

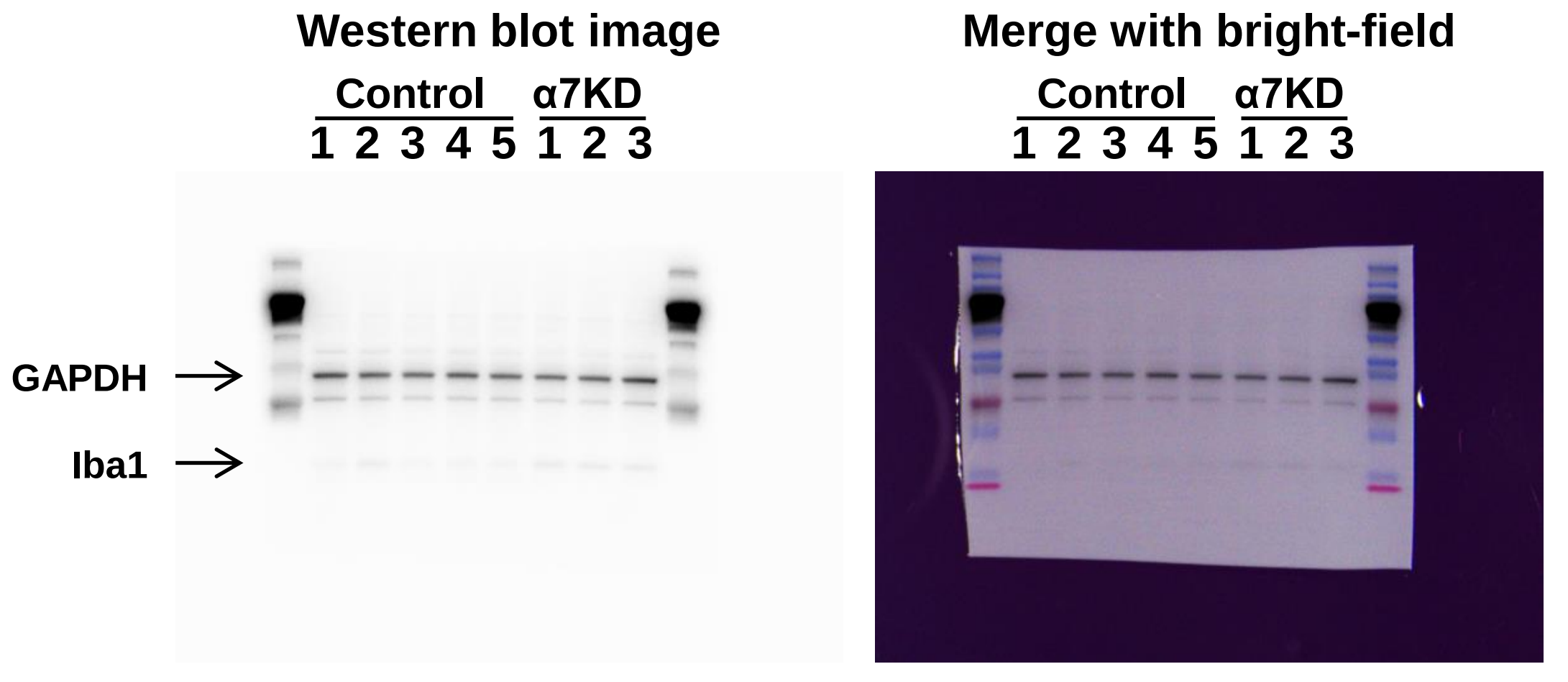

3 s exposure time:

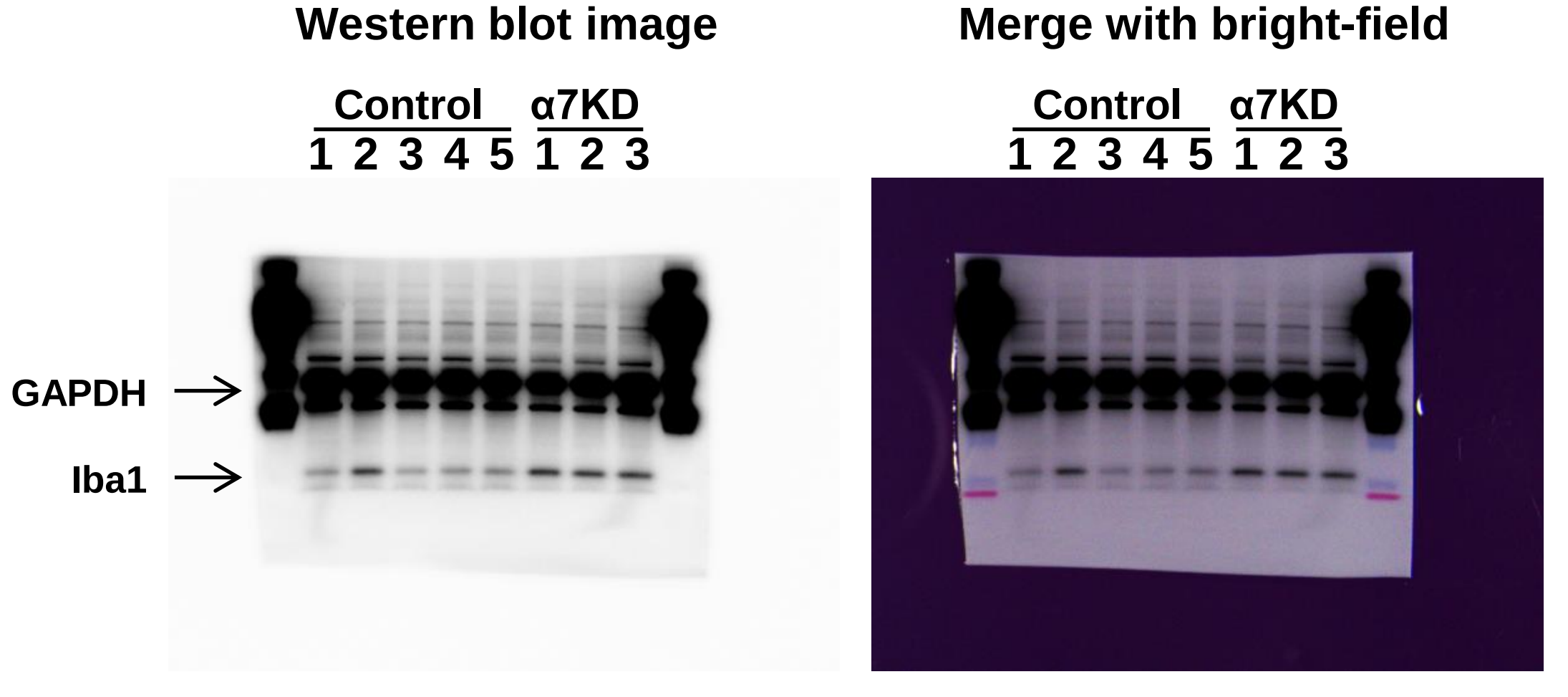

Supplement: Supplementary file 1 [file Supplementaryfile1.pdf]
